# The role of vesicle trafficking genes in osteoblast differentiation and function

**DOI:** 10.1038/s41598-023-43116-8

**Published:** 2023-09-26

**Authors:** Hui Zhu, Yingying Su, Jamie Wang, Joy Y. Wu

**Affiliations:** Division of Endocrinology, Stanford University School of Medicine, Stanford, CA USA

**Keywords:** Cell biology, Endocrinology

## Abstract

Using Col2.3GFP transgenic mice expressing GFP in maturing osteoblasts, we isolated Col2.3GFP^+^ enriched osteoblasts from 3 sources. We performed RNA-sequencing, identified 593 overlapping genes and confirmed these genes are highly enriched in osteoblast differentiation and bone mineralization annotation categories. The top 3 annotations are all associated with endoplasmic reticulum and Golgi vesicle transport. We selected 22 trafficking genes that have not been well characterized in bone for functional validation in MC3T3-E1 pre-osteoblasts. Transient siRNA knockdown of trafficking genes including *Sec24d*, *Gosr2*, *Rab2a*, *Stx5a*, *Bet1*, *Preb*, *Arf4*, *Ramp1*, *Cog6* and *Pacs1* significantly increased mineralized nodule formation and expression of osteoblast markers. Increased mineralized nodule formation was suppressed by concurrent knockdown of *P4ha1* and/or *P4ha2*, encoding collagen prolyl 4-hydroxylase isoenzymes. MC3T3-E1 pre-osteoblasts with knockdown of *Cog6*, *Gosr2*, *Pacs1* or *Arf4* formed more and larger ectopic mineralized bone nodules in vivo, which was attenuated by concurrent knockdown *P4ha2*. Permanent knockdown of *Cog6* and *Pacs1* by CRISPR/Cas9 gene editing in MC3T3-E1 pre-osteoblasts recapitulated increased mineralized nodule formation and osteoblast differentiation. In summary, we have identified several vesicle trafficking genes with roles in osteoblast function. Our findings provide potential targets for regulating bone formation.

## Introduction

Osteoblasts are major bone forming cells. Osteoblast dysregulation may cause inadequate or excessive mineralization of bones or ectopic calcification. Identification of novel genes and signaling pathways associated with osteoblast differentiation and function may lead to better understanding of bone development, homeostasis, and regeneration. Osteoblasts synthesize and secrete large amounts of type I collagen into the extracellular matrix that is subsequently mineralized to provide mechanical support and scaffolding for muscle attachment^1^. Proteins destined for secretion are synthesized in the endoplasmic reticulum (ER), then transported via vesicles to the Golgi apparatus, then in turn to the plasma membrane. During this process vesicle budding, cargo selection, transportation, and docking to destination compartments (such as the plasma membrane) are regulated by vesicle trafficking proteins such as Coat Protein Complex II (COPII) components, vesicular/target membrane proteins (v-SNARE/t-SNARE) and the Rab family of small GTPases^2^. In osteoblasts, mutations in COPII coat subunits (e.g. Sec23A and Sec24d) that regulate budding of collagen-containing vesicles from the ER are associated with skeletal defects in patients with cranio-lenticulo-sutural dysplasia^3^ and osteogenesis imperfecta (OI)^4–6^, respectively. A mutation variant of *CREB3L1*, which encodes ER-localized old astrocyte specifically induced substance (OASIS) has also been shown to be involved in altered cellular secretion in OI patients^7^.

In this study, we performed RNA sequencing of Col2.3GFP^+^ enriched osteoblasts, in which GFP expression marks mature osteoblasts^8^, and identified several vesicle trafficking genes that are involved in osteoblast differentiation and mineralization. Disruption of their expression in MC3T3-E1 pre-osteoblasts increased osteoblast differentiation and mineralized nodule formation, with enhanced collagen modification by prolyl 4-hydroxylase. Our study provides novel insight into the role of vesicle traffic proteins in osteoblast differentiation and mineralized nodule formation.

## Results

### Genes involved in vesicle trafficking are enriched in transcriptomes of mature osteoblasts

**Figure 1:**
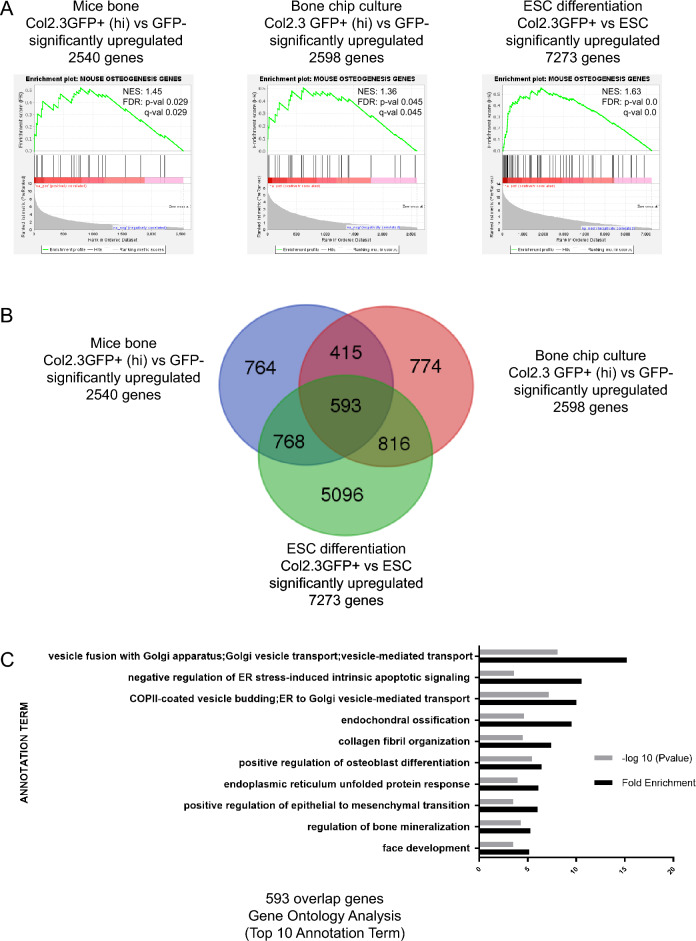
Genes involved in vesicle trafficking are enriched in transcriptomes of mature osteoblasts. (**A**) Gene set enrichment analysis (GSEA) shows significantly upregulated genes in Col2.3GFP^+^ population are enriched in osteogenesis gene set. (**B**) 593 overlapping genes that highly enriched in Col2.3GFP^+^ osteoblasts from all three resources are identified. (**C**) Gene Ontology Analysis shows that the top 3 annotation terms are all associated with ER and Golgi vesicle transport.

In Col2.3GFP transgenic mice that express green fluorescent protein (GFP) under the control of the 2.3 kb Col1a1 promoter, Col2.3GFP is expressed in maturing osteoblast lineage cells^8^. We reasoned that genome-wide gene expression profiling of the Col2.3GFP^+^ population might identify novel genes and signaling pathways critical for osteoblast function. We isolated Col2.3GFP^+^ enriched osteoblasts from three sources: (1) freshly isolated mouse bone GFP^+^ (lo) and GFP^+^ (hi) populations from long bones of Col2.3GFP transgenic mice, which represent early and late stage (mature) osteoblasts, respectively^9,10^; (2) GFP^+^ (lo) and GFP^+^ (hi) populations isolated from osteogenic differentiation of bone chips from Col2.3GFP transgenic mice^11^; and (3) GFP^+^ osteoblasts derived from osteogenic differentiation of mouse Col2.3GFP embryonic stem cell (ESC) lines^9^. We performed RNA-sequencing and confirmed that significantly upregulated genes in Col2.3GFP^+^ populations are enriched in the osteogenesis gene set (obtained from Qiagen, which includes 82 genes related to osteogenic differentiation) and in osteoblast differentiation, bone development, bone mineralization and ossification annotation categories (Fig. 1A and S1). We reasoned that overlapping genes expressed by Col2.3GFP^+^ populations from three sources of osteoblasts would further select for osteogenic candidate genes. We identified 593 overlapping genes that were highly enriched in Col2.3GFP^+^ osteoblasts from all three sources (Fig. 1B). Gene Ontology analysis reveals that the top 10 annotation terms include endochondral ossification, collagen fibril organization, positive regulation of osteoblast differentiation, and regulation of bone mineralization. Interestingly, the top 3 annotation terms are vesicle fusion with Golgi apparatus/Golgi vesicle transport; negative regulation of ER stress-induced intrinsic apoptotic signaling; and COPII-coated vesicle budding/ER to Golgi vesicle-mediated transport, all of which are associated with ER and Golgi vesicle transport (Fig. 1C). The endoplasmic reticulum unfolded protein response is also among the top 10 annotation terms (Fig. 1C). These gene annotations prompted us to focus on the function of vesicle trafficking genes in osteoblast cells.

### Transient knockdown of vesicle trafficking genes increases MC3T3-E1 pre-osteoblast osteogenic differentiation and mineralized nodule formation

**Figure 2:**
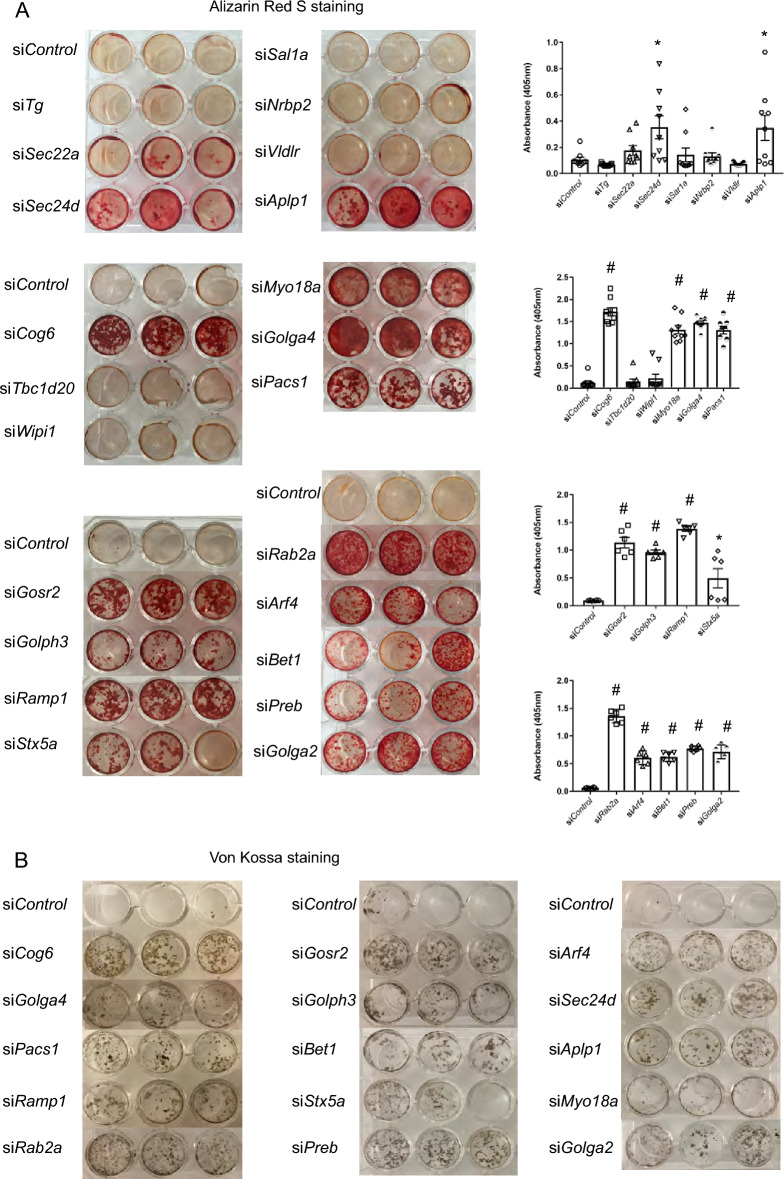
Transient knockdown of vesicle trafficking genes increases mineralized nodule formation in MC3T3-E1 osteogenic differentiation. (**A**) Ten days after osteogenic differentiation, representative Alizarin red S (**A**) and Von Kossa staining (**B**) images in control and trafficking gene knockdown MC3T3-E1 osteoblast differentiation. All data represent with scatter plot with mean ± SD (N = 6–9; multiple *t* test; *P < 0.05; **P < 0.01; ^#^*P* < 0.001; relative to si*Control*).

**Figure 3:**
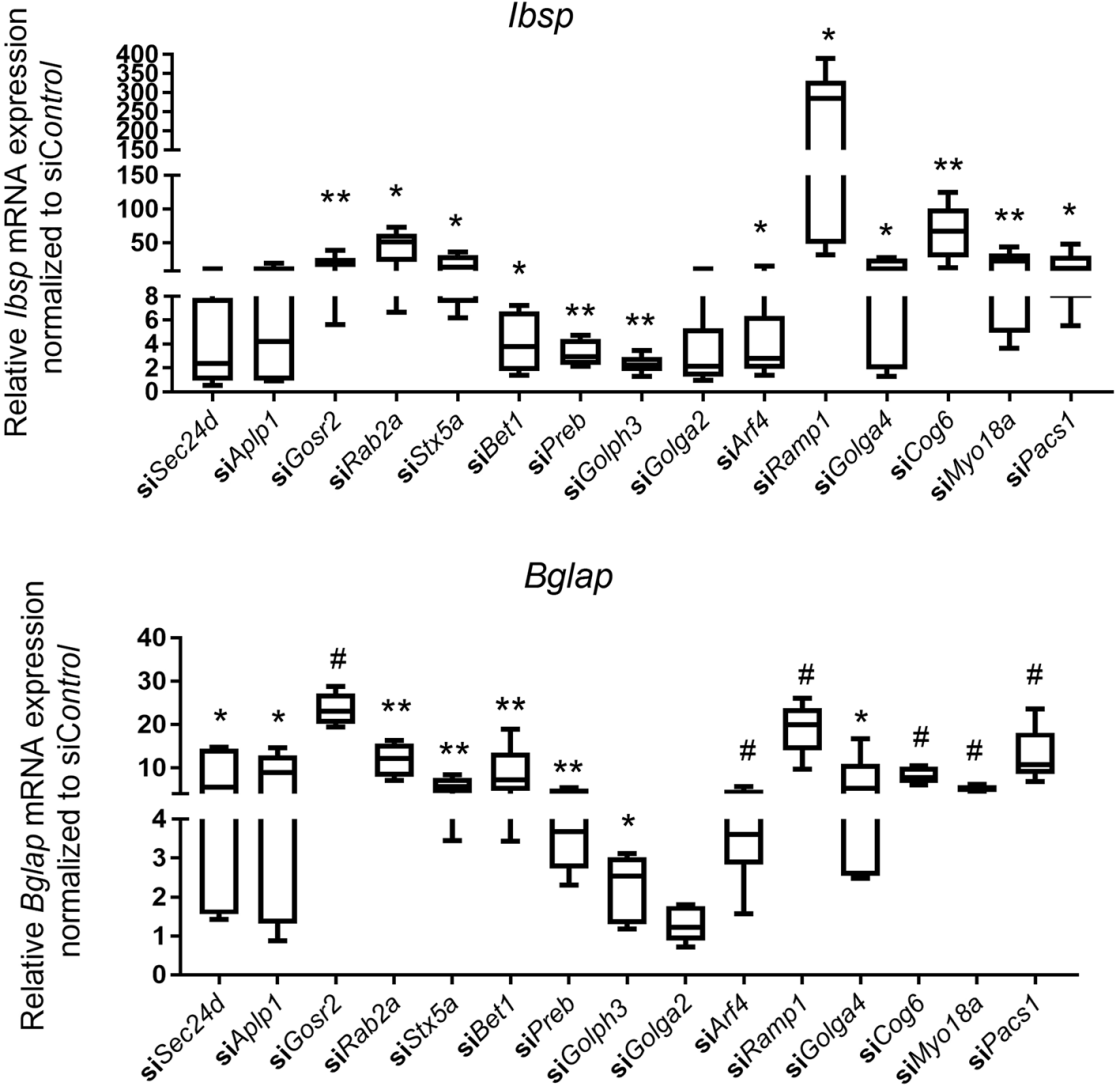
Transient knockdown of vesicle trafficking genes increases osteoblast marker expression in MC3T3-E1 osteogenic differentiation. Ten days after osteogenic differentiation, relative *Ibsp* and *Bglap* mRNA expression in control and trafficking gene knockdown MC3T3-E1 osteoblast differentiation were analyzed by qRT-PCR. Y axes show relative *Ibsp* and *Bglap* mRNA expression in trafficking gene knockdown normalized to si*Control*. All data represent with box-whiskers plot (N = 6–9; multiple *t* test; *P < 0.05; **P < 0.01; ^#^*P* < 0.001; relative to si*Control*).

A total of 57 vesicle trafficking genes are included in the top 3 annotation terms (Supplementary File: Vesicle trafficking gene lists). We selected 22 trafficking genes that have not been well characterized in bone with relatively high expression reads to perform siRNA knockdown in MC3T3-E1 pre-osteoblast cells. Interestingly we found that knockdown of 15 trafficking genes (*Sec24d*, *Aplp1*, *Gosr2*, *Rab2a*, *Stx5a*, *Bet1*, *Preb*, *Golph3*, *Golga2*, *Arf4*, *Ramp1*, *Golga4*, *Cog6*, *Myo18a* and *Pacs1*) significantly increased mineralized nodule formation by Alizarin Red S staining (Fig. 2A), confirmed by Von Kossa staining (Fig. 2B). Alkaline phosphatase activity was mildly increased by knockdown of several trafficking genes (Supplementary Fig. S2). Expression of osteoblast gene markers (*e.g*., *Ibsp* and/or *Bglap*) was also significantly increased with knockdown of these 15 trafficking genes, except for *Golga2* (Fig. 3). Knockdown efficiency was confirmed by qPCR (Supplementary Fig. S3). mRNA levels of these trafficking genes increased during osteogenic differentiation (Supplementary Fig. S4), consistent with a potential role in osteoblast differentiation and mineralized nodule formation.

Osteoblasts secrete large amounts of collagen, especially type I collagen, into the extracellular matrix where they assemble into fibers forming a scaffold for the deposition of hydroxyapatite. We examined collagen gene expression in cells with knockdown of trafficking genes. With the exception of *Preb*, *Golga2*, *Golga 4*, and *Myo18a* knockdown in which *Col1a1*, *Col1a2*, *Col4a1* or *Col4a2* mRNA levels significantly increased, knockdown of most trafficking genes did not significantly increase expression of *Col1a1, Col1a2*, *Col4a1* and *Col4a2* (Supplementary Fig. S5).

### Expression of prolyl 4-hydroxylase subunits increases with vesicle trafficking gene knockdown

**Figure 4:**
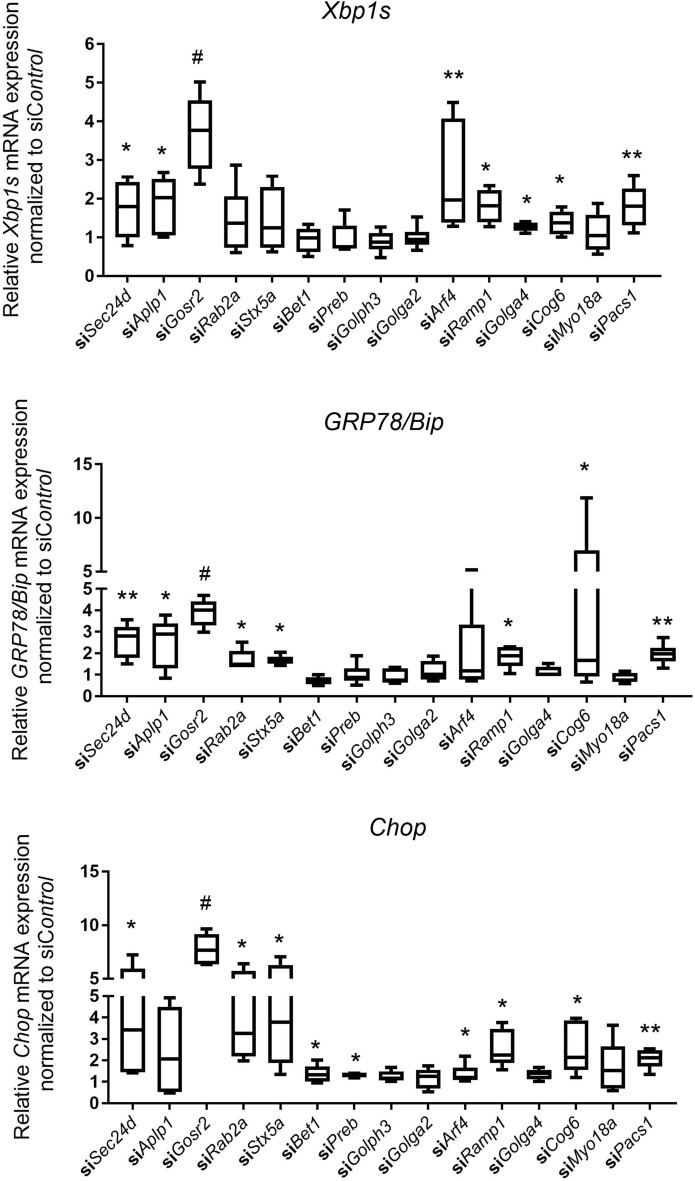
Transient knockdown of vesicle trafficking genes increases ER stress sensor gene expression in MC3T3-E1 osteogenic differentiation. Ten days after osteogenic differentiation, relative *Xbp1s*, *GRP78/Bip* and *Chop* mRNA expression in control and trafficking gene knockdown MC3T3-E1 cell differentiation were analyzed by qRT-PCR. Y axes show relative *Xbp1s*, *GRP78/Bip* and *Chop* mRNA expression in trafficking gene knockdown normalized to si*Control*. All data represent with box-whiskers plot (N = 6–9; multiple *t* test; *P < 0.05; **P < 0.01; ^#^*P* < 0.001; relative to si*Control*).

Since these trafficking genes encode ER- and/or Golgi-associated proteins that participate in vesicular transport, we examined whether knockdown of these trafficking genes affected ER homeostasis. We found that expression of at least one of the ER stress sensors (*Xbps1*, *GRP78/Bip* and *Chop*) was significantly increased with knockdown of *Sec24d*, *Aplp1*, *Gosr2*, *Rab2a*, *Stx5a*, *Bet1*, *Preb*, *Arf4*, *Ramp1*, *Golga4*, *Cog6* or *Pacs1* (Fig. 4). Disruption of ER homeostasis may lead to accumulation of misfolded proteins and the unfolded protein response^12,13^.

**Figure 5:**
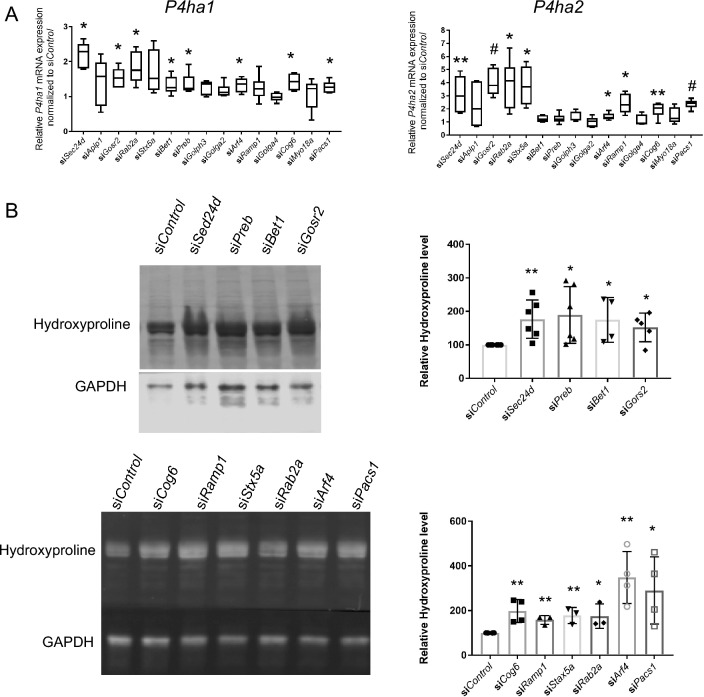
Expression of prolyl 4-hydroxylase subunit alpha *P4ha* gene and hydroxyproline protein level increase with vesicle trafficking gene knockdown in MC3T3-E1 osteogenic differentiation. (**A**) Ten days after osteogenic differentiation, relative *P4ha1* and *P4ha2* mRNA expression in control and trafficking gene knockdown MC3T3-E1 cell differentiation were analyzed by qRT-PCR. Y axes show relative *P4ha1* and *P4ha2* mRNA expression in trafficking gene knockdown normalized to si*Control*. All data represent with box-whiskers plot (N = 6–9; multiple *t* test; *P < 0.05; **P < 0.01; ^#^*P* < 0.001; relative to si*Control*). (**B**) Ten days after osteogenic differentiation, Western-blot analysis shows Hydroxyproline and GAPDH protein expression in control and trafficking gene knockdown MC3T3-E1 cell differentiation. Full gel blots are included in Supplementary Fig. S11. All data represent with scatter plots with mean ± SD (N = 3–6; multiple *t* test; *P < 0.05; **P < 0.01; ^#^*P* < 0.001; relative to si*Control*).

Hypermineralization is a hallmark of some forms of osteogenesis imperfecta (OI), the result of collagen over-modification during prolonged helical folding resulting in increased space for mineral crystal deposition between collagen molecules^14^. It has also been shown that metabolically regulated collagen modification (*e.g*., proline and lysine hydroxylation on collagen) renders the cartilaginous matrix more resistant to protease mediated degradation and thereby increases bone mass^15^. We examined expression of genes encoding several collagen-modifying enzymes including collagen prolyl 3-hydroxylase (P3H), prolyl 4-hydroxylase subunit alpha (P4HA, which forms tetramers with β subunit protein disulfide isomerase (PDI)), and lysine hydroxylases (PLOD) in MC3T3-E1 pre-osteoblast cells with knockdown of trafficking genes. Interestingly, we found that expression of at least one of collagen modifying isoenzyme was significantly increased with knockdown of 12 trafficking genes (*Sec24d*, *Aplp1*, *Gosr2*, *Rab2a*, *Stx5a*, *Bet1*, *Preb*, *Arf4*, *Ramp1*, *Cog6*, *Myo18a* and *Pacs1*) (Fig. 5A, Supplementary Fig. S6 and S7). Expression of one or both collagen prolyl 3-hydroxylase (*P3h*) isoenzymes was significantly increased with knockdown of *Aplp1*, *Arf4*, *Myo 18a* and *Pacs1*. Expression of at least one of the lysine hydroxylases (*Plod*) isoenzymes was significantly increased with knockdown of *Sec24d*, *Gosr2*, *Bet1*, *Arf4* and *Pacs1*. Notably, expression of *P4ha1* and/or *P4ha2,* which encode prolyl 4-hydroxylase subunit alpha (P4HA) was significantly increased with knockdown of 10 trafficking genes (*Sec24d*, *Gosr2*, *Rab2a*, *Stx5a*, *Bet1*, *Preb*, *Arf4*, *Ramp1*, *Cog6* and *Pacs1*). Prolyl 4-hydroxylase plays a central role in collagen synthesis as 4-hydroxyproline residues are essential for the formation of triple helical molecules in vivo^16^. Therefore, we focused on *P4ha1* and *P4ha2* for further study.

### Concurrent knockdown of *P4ha1/P4ha2* rescues increased mineralized nodule formation associated with knockdown of vesicle trafficking genes

**Figure 6:**
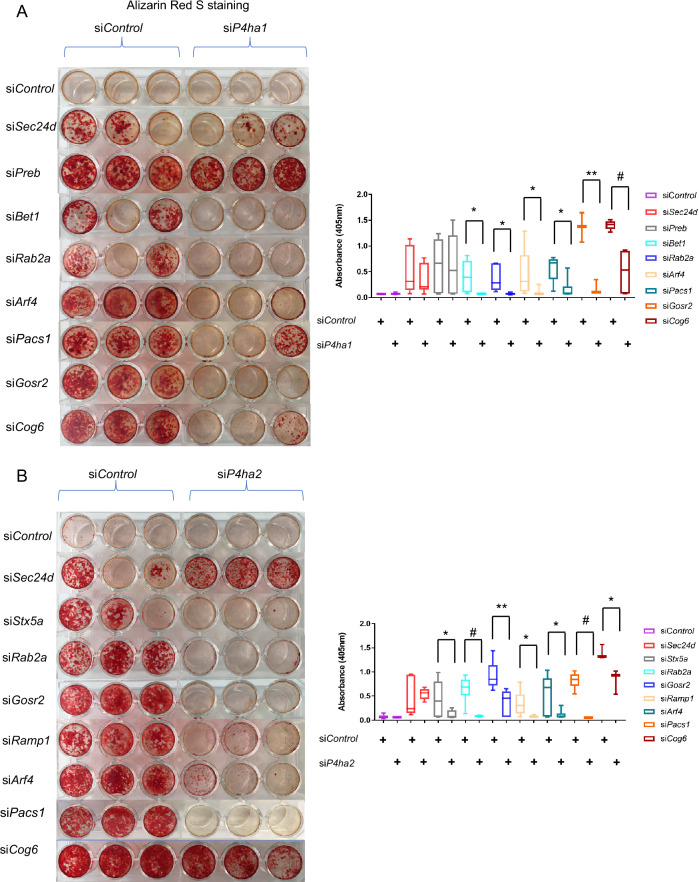
Concurrent knockdown of *P4ha1/P4ha2* rescues increased mineralized nodule formation associated with knockdown of vesicle trafficking genes. Ten days after osteogenic differentiation, representative Alizarin red S staining images and quantification analysis in control and trafficking genes with/without concurrent knockdown of *P4ha1* (**A**) and *P4ha2* (**B**) MC3T3-E1 cell differentiation. All data represent with box-whiskers plot (N = 6–9; multiple *t* test; *P < 0.05; **P < 0.01; ^#^*P* < 0.001).

We examined total intracellular hydroxyproline protein levels and found they were significantly increased with knockdown of *Sec24d*, *Gosr2*, *Rab2a*, *Stx5a*, *Bet1*, *Preb*, *Arf4*, *Ramp1*, *Cog6* and *Pacs1* trafficking genes during osteogenic differentiation (Fig. 5B). To determine whether increased *P4ha* expression contributed to increased mineralized nodule formation with knockdown of trafficking genes, we performed concurrent siRNA knockdown of *P4ha1* or *P4ha2* with *Sec24d*, *Gosr2*, *Rab2a*, *Stx5a*, *Bet1*, *Preb*, *Arf4*, *Ramp1*, *Cog6* or *Pacs1* trafficking genes. Knockdown of *P4ha1* and *P4ha2* was confirmed by significantly decreased P4HA1 and P4HA2 protein levels (Supplementary Fig. S8). Concurrent knockdown of *P4ha1* rescued the increased nodule formation phenotype associated with knockdown of *Gosr2*, *Rab2a*, *Bet1*, *Arf4*, *Cog6* and *Pacs1* trafficking genes (Fig. 6A*)*. Concurrent knockdown of *P4ha2* rescued the increased nodule formation phenotype associated with knockdown of *Gosr2*, *Rab2a*, *Stx5a*, *Arf4*, *Ramp1*, *Cog6* and *Pacs1* trafficking genes (Fig. 6B). These data suggest a role for prolyl 4-hydroxylation in the increased mineralized nodule formation results with knockdown of *Gosr2*, *Rab2a*, *Stx5a*, *Bet1*, *Arf4*, *Ramp1*, *Cog6* and *Pacs1* trafficking genes. Of note, neither osteoblast marker nor ER stress genes were significantly affected by concurrent knockdown *P4ha1* or *P4ha2* with *Gosr2*, *Rab2a*, *Stx5a*, *Bet1*, *Arf4*, *Ramp1*, *Cog6* and *Pacs1* (Supplementary Fig. S9), suggesting that prolyl 4-hydroxylation is directly involved in mineralized nodule formation rather than osteoblast differentiation and/or ER stress.

### Vesicle trafficking gene knockdown increases mineralized nodule formation in vivo

**Figure 7:**
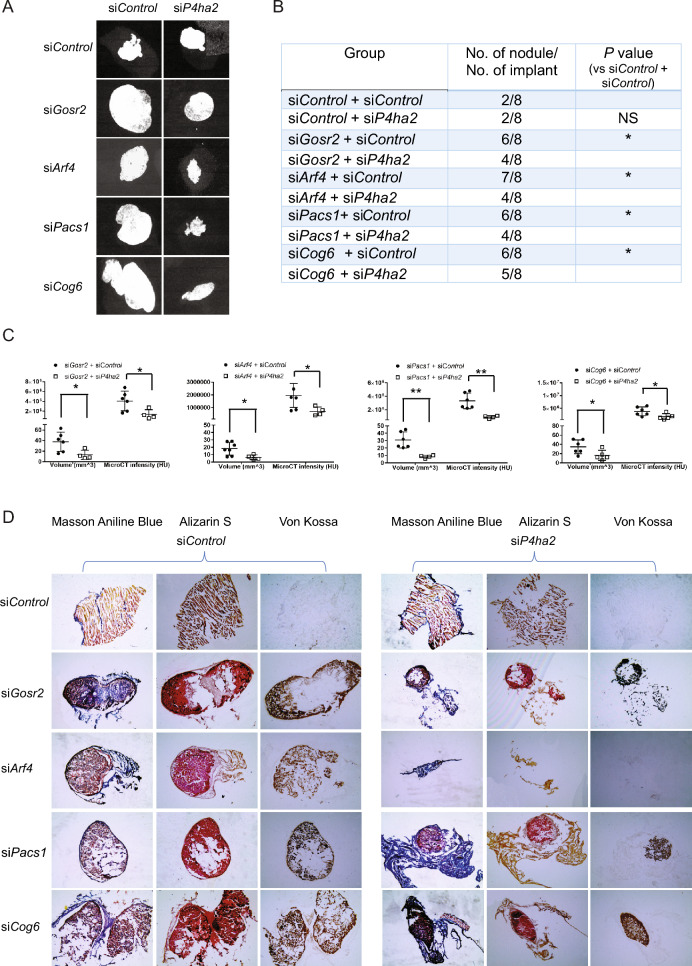
Vesicle trafficking gene depletion increases mineralized nodule formation in vivo*.* MC3T3-E1 cells transfected with Control siRNA, trafficking gene siRNA with/without *P4ha2* siRNA and Matrigel mixtures were subcutaneously injected into immunodeficient mice. Eight weeks later, mice were euthanized and dissected. (**A**) Representative micro-CT X-ray images of explanted nodules show significant mineralized structure in each cell group of implants. (**B**) Incidences of mineralized nodule formation in each group. *P < 0.05; Z proportion score. (**C**) Quantification of mineralized nodule volume and micro-CT total intensity in each group. All data represent with interleaved scatter plots with mean ± SD (multiple *t* test; *P < 0.05; **P < 0.01; ^#^*P* < 0.001). (**D**) Serial cryosections were made in each explanted cell nodules. Mineralized structures were visualized using Alizarin red S and Von Kossa staining. Newly formed mineralized structures were visualized by Aniline Blue staining.

To examine the effects of vesicle trafficking gene knockdown on mineralized nodule formation in vivo, we knocked down *Gosr2*, *Arf4*, *Cog6* and *Pacs1* (selected for the most consistency of in vitro phenotypes) with and without concurrent knockdown of *P4ha2* in MC3T3-E1 pre-osteoblasts, which were mixed with Matrigel and implanted subcutaneously into immunodeficient mice. Mice were euthanized at 8 weeks and implants were analyzed by X-ray and micro-CT. The number of mineralized nodules formed was significantly higher with knockdown of *Gosr2*, *Arf4*, *Cog6* or *Pacs1* compared to control (Fig. 7A, B). While concurrent knockdown of *P4ha2* did not significantly reduce the numbers of mineralized nodules compared to knockdown of *Gosr2*, *Arf4*, *Cog6* or *Pacs1* alone, mineralized lump volume and micro-CT total intensity were significantly decreased (Fig. 7C). We confirmed mineralization by histological analysis of nodules stained with Alizarin Red S and Von Kossa (Fig. 7D). Collectively, these data suggest that knockdown of vesicle trafficking gene *Gosr2*, *Arf4*, *Cog6* and *Pacs1* increases mineralized nodule formation in vivo in a *P4ha2*-dependent manner.

### ***Cog6***^−/−^ and ***Pacs1***^−/−^ MC3T3-E1 cells exhibit increased osteogenic differentiation and mineralized nodule formation

**Figure 8:**
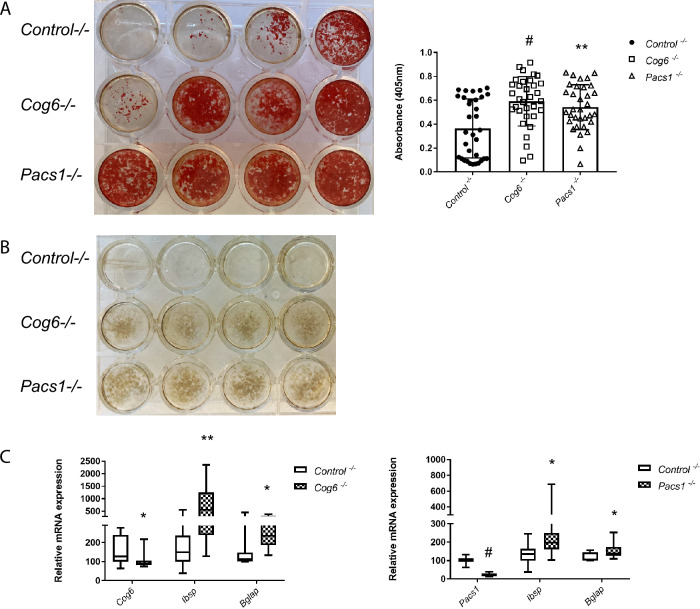
*Cog6*^**−**/**−**^ and *Pacs1*^**−**/**−**^ MC3T3-E1 cells exhibit increased osteogenic differentiation and mineralized nodule formation. *Cog6*^**−**/**−**^, *Pacs1*^**−**/**−**^ MC3T3-E1 CRISPR lines and control CRISPR line were subjected to osteogenesis for ten days. (**A**) Representative Alizarin red S staining images and quantification analysis in control and *Cog6*^**−/−**^**,** *Pacs1*^**−/−**^ CRISPR line differentiation. All data represent with scatter plots with mean ± SD (N = 33; multiple *t* test; *P < 0.05; **P < 0.01; ^#^*P* < 0.001). (**B**) Von Kossa staining in control and *Cog6*^**−/−**^**,** *Pacs1*^**−/−**^ CRISPR line differentiation. (**C**) qRT-PCR analysis shows relative *Ibsp* and *Bglap* mRNA expression in control and *Cog6*^**−/−**^**,** *Pacs1*^**−/−**^ CRISPR line differentiation. All data represent with box-whiskers plot (N = 11–13; multiple *t* test; *P < 0.05; **P < 0.01; ^#^*P* < 0.001; relative to *Control*^**−/−**^).

siRNA-mediated knockdown of trafficking genes is transient. To test the effects of permanent deletion of vesicle trafficking genes and further study the mechanisms underlying increased mineralized nodule formation, we generated loss-of-function mutations in the *Cog*6 and *Pacs1* genes used CRISPR-mediated editing. mRNA analysis confirmed the decreased mRNA in *Cog6*^**−/−**^ and *Pacs1*^**−/−**^ MC3T3-E1 CRISPR lines compared to control CRISPR line (Supplementary Fig. S10A). Deletion of *Cog6* or *Pacs1* had no effect on cell proliferation (Supplementary Fig. S10B). Congruent with transient siRNA knockdown results, *Cog6*^**−/−**^ and *Pacs1*^**−/−**^ MC3T3-E1 CRISPR lines displayed increased mineralized nodule formation compared to control CRISPR line subjected to osteogenesis (Fig. 8A and 8B). Expression of osteogenic markers *Ibsp* and *Bglap* were significantly higher compared to control CRISPR line, suggesting accelerated osteogenic differentiation (Fig. 8C). This data validates that lack of vesicle trafficking genes *Cog6* or *Pacs1* accelerated osteogenic differentiation and mineralized nodule formation in MC3T3 pre-osteoblasts.

## Discussion

By performing RNA-sequencing on three sources of Col2.3GFP^+^ maturing osteoblasts, we identified 593 overlapping genes that are enriched among osteoblasts including genes encoding osteogenic markers (e.g., *Ibsp*, *Bglap*), osteogenic transcription factors (e.g., *Runx2*, *Sp7, Dlx5*) as well as genes in which mutations have been associated with skeletal defects (e.g., *Sec24d*, *Creb3l1*). Of note, the top three gene annotation terms were all related to vesicle mediated ER/Golgi transport.

We found that transient siRNA knockdown of 15 trafficking genes (*Sec24d*, *Aplp1*, *Gosr2*, *Rab2a*, *Stx5a*, *Bet1*, *Preb*, *Golph3*, *Golga2*, *Arf4*, *Ramp1*, *Golga4*, *Cog6*, *Myo18a* and *Pacs1*) increased mineralized nodule formation and osteoblast marker gene expression. Vesicle trafficking proteins are responsible for cargo selection, transportation, and secretion^2^. Disruption of vesicle trafficking gene expression and function can lead to the unfolded protein response, delayed collagen folding, prolonged collagen transportation, and increased collagen modification. In OI type I less than 50% of collagen is produced in an environment with a normal level of lysyl hydroxylating enzymes which results in an increased enzyme to collagen ratio and subsequent higher levels of hydroxylation and glycosylation as well^14^. Also, proline hydroxylation enhances the stability of the collagen triple helices and lysine modifications increase crosslinking^16^. In OI increased hydroxylation and glycosylation of collagen leads to greater distance between collagen molecules within the fibril. As a result of this steric hinderance more crystals can be deposited within collagen fibrils, leading to hypermineralization^14^. Increased expression of ER stress markers associated with knockdown of *Gosr2*, *Rab2a*, *Arf4*, *Cog6*, *Pacs1*, *Bet1*, *Stx5a* or *Ramp1* suggest that ER homeostasis may have been disrupted. It has been shown that increased matrix deposition during endochondral ossification is associated with activation of endoplasmic reticulum stress sensors, which in turn changed the availability of the metabolic substrates and further increased gene expression levels of collagen-modifying enzymes including collagen prolyl hydroxylases, lysine hydroxylases and lysyl oxidase^15^. Therefore, one possible mechanism for the increased mineralized nodule formation seen with knockdown of trafficking genes *Gosr2*, *Rab2a*, *Arf4*, *Cog6*, *Pacs1*, *Bet1*, *Stx5a* or *Ramp1* might be due to enhanced prolyl 4-hydroxylation caused by increased *P4ha1* and/or *P4ha2* expression, which in turn increases the space available for mineral deposition among the extracellular matrix. Consistent with this mode, increased mineralized nodule formation resulting from knockdown of *Gosr2*, *Rab2a*, *Arf4*, *Cog6*, *Pacs1, Bet1, Stx5a* and *Ramp1* was associated with increased *P4ha1* and/or *P4ha2* expression, and double knockdown of these trafficking genes and *P4ha1* and/or *P4ha2* reduced mineralized nodule formation. In an ectopic bone formation model, knockdown of *Gosr2*, *Arf4*, *Cog6* and *Pacs1* increased mineralized nodule formation in vivo, while concurrent knockdown of *P4ha2* rescued this phenotype.

Additional collagen-modifying enzymes may also play a role. In particular, expression of prolyl 3-hydroxylase genes *P3h1* and/or *P3h2* was increased by knockdown of *Aplp1*, *Arf4*, *Myo18a* or *Pacs1* while expression of the lysine hydroxylase gene *Plod* was increased by knockdown of *Sec24d*, *Gosr2*, *Bet1*, *Arf4* or *Pacs1*. Of the 15 vesicle trafficking genes we identified that exhibited increased mineralized nodule formation upon transient knockdown, knockdown of *Golph3*, *Golga2 and Golga4* was not associated with increased *P4ha*, *P3h*, and *Plod* expression. Transient knockdown of trafficking genes was also associated with increased expression of osteoblast marker genes, suggesting enhanced osteoblast differentiation. Strikingly, double knockdown *P4ha1* or *P4ha2* with *Gosr2*, *Rab2a*, *Stx5a*, *Bet1*, *Arf4*, *Ramp1*, *Cog6* and *Pacs1* did not rescue increased osteoblast marker gene expression, suggesting that regulation of osteoblast differentiation by trafficking genes may be an independent mechanism contributing to mineralized nodule formation. Further investigation and elucidation of signal pathways and mechanisms involved in trafficking gene regulating osteoblast differentiation are important.

The specific function of each trafficking gene in osteoblast differentiation and function remains to be determined. Since siRNA-mediated knockdown is transient, we performed CRISPR/Cas-9-mediated gene editing to permanently knock down *Cog6*^**−/−**^ and *Pacs1*^**−/−**^ in MC3T3-E1 pre-osteoblasts and found that this similarly increases mineralized nodule formation and osteoblast marker gene expression in vitro*.*

We have further demonstrated that MC3T3 pre-osteoblasts with transient knockdown of *Gosr2*, *Arf4*, *Cog6* and *Pacs1* form more and larger ectopic bone nodules in vivo. However, conditional knockout models will be required to examine the role of trafficking genes in endogenous bone formation, and on bone mass, strength and quality. Cog6 (Component of Oligomeric Golgi Complex 6) belongs to the COG family which is an evolutionally conserved Golgi-associated tethering complex^17^. Cog6 is directly involved in both intra-Golgi and endosome to Golgi retrograde transport^18^. Interestingly, a recent mouse genome-wide association study (GWAS) identified a bone mineral density-associated locus containing *Cog6* and showed that *Cog6* was highly expressed both in bone and osteoblasts^19^. Pacs1 (Phosphofurin acidic cluster sorting protein 1) is a member of the trans-Golgi network (TGN) membrane protein family and directs the TGN localization of furin^20^. Pacs1 is critical for protein trafficking in vertebrates by binding to diverse cargo and trafficking machinery, including the adaptor protein-1 complex, to ensure the proper cytoplasmic localization of numerous proteins^21–23^. A function for Pacs1 in osteoblasts or bone has not been reported. Gosr2 (Golgi SNAP Receptor Complex Member 2) belongs to a group of SNARE (soluble N-ethylmaleimide-sensitive factor attachment protein receptor) proteins that are essential for budding, docking and fusion of COPII-coated vesicles during ER-Golgi membrane trafficking^24,25^. *Gosr2* gene was identified as a causative gene for progressive myoclonus epilepsy with distinct clinical features include scoliosis^26^. It has also been shown that Gosr2 forms complex with Stx5 and Bet1 and the complex are responsible for fusion of endoplasmic reticulum derived vesicles with the ER-Golgi intermediate compartment and the cis-Golgi^24,25,27^. Similar to knockdown of *Gosr2*, we also found increased osteogenic differentiation and mineralized nodule formation with knockdown of *Stx5, Bet1* and* Rab2a* in MC3T3-E1 pre-osteoblasts. Both Bet1 and Rab2a are members of the GTPase family and are involved in post-endocytic trafficking of membrane-bound MT1-MMP (MMP14, membrane type 1-matrix metalloproteinase), an essential metalloprotease for matrix remodeling, invasion, pericellular collagenolysis and skeletal/extraskeletal connective tissue modeling^28–30^. Arf4 (ADP Ribosylation Factor 4) is another vesicle trafficking protein belonging to GTPase family and functions an ADP-ribosyltransferase^31^. ARF4 regulates ciliary protein trafficking, dysfunction of which is a known cause of human genetic diseases and syndromic disorders known as ciliopathies^31–33^. Both *Rab2a* and *Arf4* are highly expressed in osteoblasts and bone (data from BioGPS, http://biogps.org/)^34^.

In summary, we identified a set of vesicle trafficking genes that are highly expressed in Col2.3GFP^+^ osteoblast lineage cells and play a role in osteoblast differentiation and mineralized nodule formation. Osteoblasts synthesize, transport, and secrete large amounts of bone matrix components including collagen, which is tightly regulated. Any dysregulation would cause inadequate or excessive mineralization of bones. Elucidation of the specific function of vesicle traffic genes involving in osteoblasts function raises the possibility of developing novel therapeutics for bone diseases designed to target this process.

## Methods

### Tissue culture and osteogenic differentiation

Generation, maintenance and differentiation of mouse Col2.3GFP ESCs were performed as previously described^9^. Bone chips were harvested from Col2.3GFP transgenic mice and subjected to osteogenic differentiation as previously described^35^.

The MC3T3-E1 pre-osteoblast cell line (passage 35–45) was kindly provided by the David Feldman laboratory (Stanford University). MC3T3-E1 cells were maintained in basal medium (αMEM, no ascorbic acid, cat A1049001; Thermo Fisher Scientific, Waltham, MA) supplemented with 10% fetal bovine serum (FBS; Gemini Bio Products, CA) and 1% penicillin–streptomycin. For osteogenic differentiation, the basal medium was replaced by osteogenic medium (basal medium supplemented by 50 μg/mL ascorbic acid (Sigma, St. Louis, MO)). For mineralization assays, 10 mM β-glycerol phosphate (Sigma, St. Louis, MO) and 100 nM dexamethasone (Sigma, St. Louis, MO) were added to the osteogenic medium. Osteogenic medium was changed every three days.

### RNA interference

For transient knockdown target gene, siGENOME SMARTpool siRNA for specific target genes and non-Targeting siRNA for si*Control* were synthesized and purchased from Dharmacon, Inc. (Lafayette, CO). Cells were plated at a concentration of 1 X 10^4^ cells/well in 24-well plates and transfected with the siRNA using Lipofectamine RNAiMax transfection reagent (Thermo Fisher Scientific) according to the manufacturer’s instructions on the following day after plating. 2.5 µl of 10 µM siRNA duplexes were mixed with 47.5 µl Opti-MEM (Thermo Fisher Scientific) and combined with 1 µl/well of transfection reagent and 49 µl Opti-MEM to a total volume of 100 µl and incubated for 20 min. After incubation, 100 µl siRNA-lipid complex were transfected to the adherent cells. Osteogenic differentiation was initiated with osteogenic medium on the second day after transfection. Transfected cells were analyzed at different time points.

### Von Kossa, Alizarin Red S and alkaline phosphatase staining

For Von Kossa staining, cells were rinsed twice with PBS and fixed in 10% formaldehyde for 10 min, then washed with double distilled water (DDW). Cells were incubated in 5% silver nitrate solution under UV light for 30 min, washed with DDW, rinsed with 5% sodium thiosulfate for 5 min to remove unreacted silver, washed again with DDW and stored in PBS. For Alizarin Red S staining cells were fixed and washed, stained with 2% Alizarin Red S solution for 20 min, then washed with DDW to remove unincorporated excess dye. For quantification of Alizarin Red S staining, following the wash in DDW cells were incubated in 10% Acetic acid with shaking for 30 min at room temperature. Cells were collected by cell scraper and transferred to microcentrifuge tubes, heated for 10 min at 85 °C, and then put on ice for 5 min. After centrifugation at 20,000 g for 15 min the supernatant was transferred to a new microcentrifuge tube. Upon addition of 10% Ammonium hydroxide, 50 µl aliquot was transferred to a microplate reader well and absorbance was measured at 405 nm. Alkaline phosphatase staining was performed by using Stemgent^®^ Alkaline Phosphatase Staining Kit II (Stemgent, San Diego, CA) according to the manufacturer’s instructions.

### Quantitative reverse transcription PCR (qPCR) and RNA-sequencing

RNA was extracted by using PureLink^®^ RNA Mini Kit (Invitrogen, Carlsbad, CA). RNA concentration and purity were measured by NanoDrop (Thermo Scientific, Wilmington, DE) and then used for reverse transcription using iScript Kit (Bio-Rad, Hercules, CA). Quantitative RT-PCR (qPCR) was performed following standard methods. Primer sequences are provided in Supplementary Table. mRNA levels were normalized to of the levels of *β-Actin*. Relative mRNA levels of trafficking gene knockdown cells were then normalized to si*Control*. PCR was performed in triplicate for each sample, and 3 independent experiments were carried out^11^.

For RNA-sequencing (RNA-seq), the integrity of extracted RNA was assayed by on-chip electrophoresis (Agilent Bioanalyzer) and only samples with a high RNA integrity (RIN) value were used for RNA-seq^11^. Poly-A mRNA was purified using Dynabeads^®^ mRNA Purification Kit (Invitrogen) according to the manufacturer’s instructions. RNA sequencing libraries were prepared using the NEBNext^®^ Ultra™ II DNA Library Prep Kit for Illumina (New England Biolabs, Ipswich, MA) according to the manufacturer’s instructions. Library quality was verified using the Agilent High Sensitivity DNA Kit on Agilent’s 2100 Bioanalyzer. For each library, an average of 420 bp fragments were sequenced using paired end reads (2 × 100 bp) on the Illumina HiSeq 2500 platform (Stanford Personalized Medicine Sequencing Core), with an average of 30 million reads per sample. Paired end sequencing reads (100 bp) were generated and aligned to mouse reference sequence NCBI Build 37/mm9 with the STAR (v2.4.2a) algorithm^36^. Normalization of RefSeq annotated genes, expression level and differential expression analysis were performed using the Bioconductor package DESeq2 in R (Version 3.2.2)^37^. Genes with False Discovery Rate (FDR) < 0.05 were defined as differentially expressed genes. Gene ontology analysis was performed using DAVID *(david.abcc.ncifcrf.gov)* and The Gene Ontology (GO) Consortium (http://geneontology.org/). For Gene Set Enrichment Analysis (GSEA, Broad Institute), gene set associated with osteogenesis were obtained from www.qiagen.com (Qiagen, Germany)^38^. All transcriptome raw data are publicly available in GEO (accession number GSE223192, Genome wide gene expression profiling of Col2.3GFP^+^ enriched osteoblasts).

### Western blot analysis

Cells were lysed in Radioimmunoprecipitation (RIPA) assay buffer (Sigma, St. Louis, MO), total protein was extracted, and the protein concentration was measured by Bradford Protein Assay (Bio-Rad). Protein samples were loaded on 10% SDS-PAGE gel and the separated proteins were transferred by electroblotting to polyvinylidene fluoride (PVDF) membranes (Bio-Rad). The membranes were blocked with Odyssey^®^ Blocking Buffer (LI-COR Biosciences, Lincoln, NE) and incubated with the primary antibody and secondary antibody. Immunolabeling was detected by using Odyssey^®^ CLx system (LI-COR Biosciences). The following primary antibodies were used: Anti-Hydroxyproline antibody (ab37067, Abcam; 1:1000 dilution); Anti-P4HA1 antibody (ab59497, Abcam; 1:500 dilution); Anti-P4HA2 antibody (A4262, ABclonal Inc.; 1:500 dilution); Anti-GAPDH antibody (sc-32233, Santa Cruz Biotechnology, Inc.; 1:1000 dilution).

### Subcutaneous implantation

Seven-week-old male CD-1 Nude Mice Crl:CD1-Foxn1nu purchased from Charles River (Wilmington, MA, USA) were housed in Innovive recyclable individually ventilated cages in a designated pathogen-free area facility and fed irradiated mouse chow and autoclaved water. The Veterinary Service Center (VSC) at Stanford University provides laboratory animal care and is administered by the Department of Comparative Medicine. The laboratory animal care program at Stanford University is fully accredited by the Association for Accreditation and Assessment of Laboratory Animal Care (AAALAC). All animal surgeries were approved by the Stanford University Administrative Panel on Laboratory Animal Care (APLAC). All methods were performed in accordance with relevant guidelines and regulations. This study is reported in accordance with ARRIVE guidelines (https://arriveguidelines.org). MC3T3-E1 cells were transfected siRNA and subjected to osteogenic differentiation for seven days. After anesthesia using inhaled isoflurane, cells (100 µl) were mixed with equal volume (100 µl) of Matrigel and then subcutaneously implanted into the back of 7-week-old male CD-1 Nude Mice Crl:CD1-Foxn1^nu^. Each implantation was conducted by injection of 1 × 10^6^ cells and Matrigel mixture using 25G needles. Four implantations were injected on each side of the spine. Nodules were explanted after 12 weeks and analyzed by X-ray, microCT and histology. We implanted four single trafficking gene-knockdown samples in one spine side of mouse and implanted four trafficking gene and *P4ha2* double knockdown samples on the other spine side of the mouse to minimize mouse variation.

### Micro-computed tomography and histological analysis

Mineralization of subcutaneous implants was monitored by micro-computed tomography (micro-CT) using the vivaCT 40 Preclinical MicroCT Scanner (SCANCO Medical, AG)^11^. For small nodules formed after subcutaneous implantation, scanning was conducted with X-ray setting at Energy/intensity: 70 kVp,114 μA, 8w; with CT-Scan setting at high resolution, 21.5 mm FOV (field of view), 10.5 μm voxel size and 618 slices. The region of interest was selected to completely cover the nodules. X-ray images were analyzed by Inveon™ Research Workplace 4.2 software (IRW 4.2, Siemens Healthcare GmbH, Germany)^11^. For histological analysis, tissue samples were harvested and fixed for 24 h in 10% neutral-buffered formalin followed by incubation in 30% sucrose for 24 hours^11^. Tissue samples were embedded in SCEM embedding medium (Section-lab, Japan). 8-μm serial sections were made on a cryofilm (Section-lab, Japan) using tape-stabilized sectioning method for histology analysis. Aniline Blue staining was performed according to Masson’s trichrome method (Newcomer Supply, Inc., Middleton, WI)^11^. Von Kossa and Alizarin Red S staining were performed on sectioned slides^11^. Images were acquired by an EVOS FL Cell Imaging System (Live Technologies, South San Francisco, CA)^11^.

### CRISPR/Cas9-mediated gene editing in MC3T3-E1 pre-osteoblast cells

*Cog6*^**−/−**^ and *Pacs1*^**−/−**^ MC3T3-E1 cells were generated by CRISPR-Cas9 gene editing technology as previously described^39^. Briefly, guide RNA sequences targeting *Cog6* (5′-CGGACTCGAAGAAATTTACG-3′), *Pacs1* (5′-CAGTGACGACCCATTGCAT-3′) and non-targeting control (5′-GAACTCGTTAGGCCGTGAAG-3′) were cloned into lentiCRISPR v2 plasmid (Addgene; cat. no. 52961). Lentivirus was generated and harvested in 293 T cells by transfecting cells with lentiviral vectors along with the packaging vectors psPAX2 and pMAD2.G (Addgene; cat. no. 16620 and 16,619, respectively) as previously described^11^. MC3T3-E1 cells were transduced with the lentivirus and selected with puromycin containing MEM (2 µg/ml) for ten days. *Cog6*^**−/−**^ and *Pacs1*^**−/−**^ pooled knockout cell lines were analyzed by qRT-PCR to obtain clones with permanent depletion of the gene product. CRISPR line proliferation rate was assessed by Cell Proliferation Assay (MTS) according to the manufacturer’s instructions (cat NO: G3582; Promega, Madison, WI).

### Statistical analysis

Statistical analysis was performed using Microsoft Excel and GraphPad Prism 6 (GraphPad Software, San Diego, CA). Quantitative data are presented as scatter or interleaved scatter plots with mean ± SD, or box-whiskers plots with whiskers representing minimum and maximum values and box representing 1st to 3rd quartiles.

Shapiro–Wilk normality tests were performed to test whether data were normally distributed. If Shapero–Wilk tests were not satisfied, nonparametric tests were used. Statistical significance was assessed by multiple *t* test followed by correction for multiple comparisons using the Holm–Sidak method.

## Supplementary Information

Supplementary Information 1.

Supplementary Information 2.

## Data Availability

The raw data required to reproduce these findings are available in the Open Data sectionGEO (accession number GSE223192, Genome wide gene expression profiling of Col2.3GFP^+^ enriched osteoblasts).
