# Correction: The role of vesicle trafficking genes in osteoblast differentiation and function

**DOI:** 10.1038/s41598-025-03473-y

**Published:** 2025-05-28

**Authors:** Hui Zhu, Yingying Su, Jamie Wang, Joy Y. Wu

**Affiliations:** Division of Endocrinology, Stanford University School of Medicine, Stanford, CA USA

Correction to: *Scientific Reports* 10.1038/s41598-023-43116-8, published online 26 September 2023

The original version of this Article contained error in Figure 7A, where the upper right (si*Control*/si*P4ha2*) and lower right (si*Cog6*/si*P4ha2*) panels were duplicated.

The original Figure 7 and accompanying legend appear below.

**Fig. 7:**
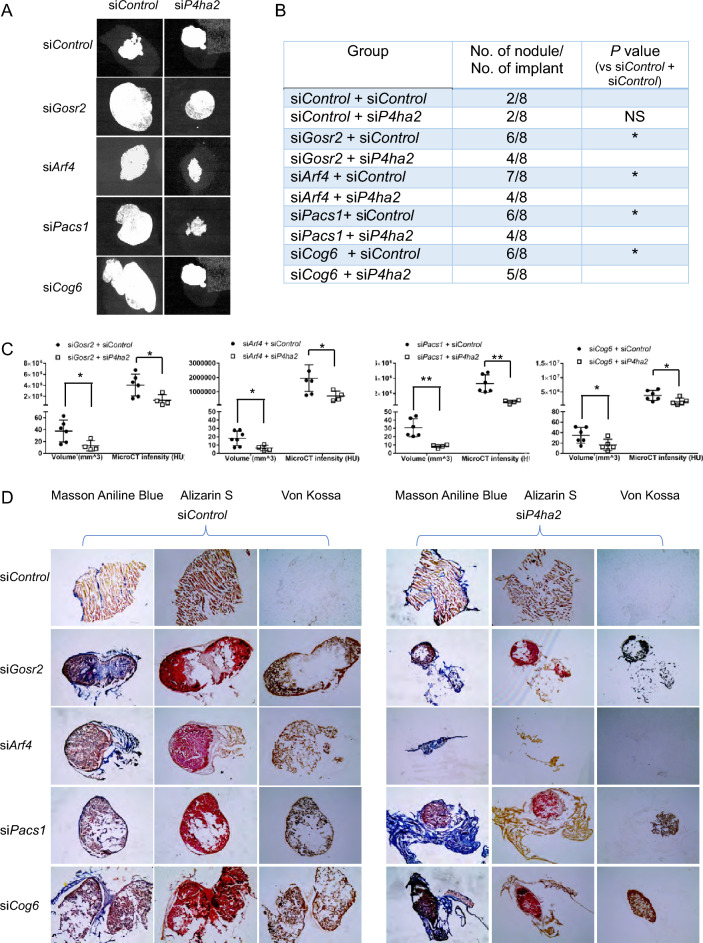
Vesicle trafficking gene depletion increases mineralized nodule formation in vivo*.* MC3T3-E1 cells transfected with Control siRNA, trafficking gene siRNA with/without *P4ha2* siRNA and Matrigel mixtures were subcutaneously injected into immunodeficient mice. Eight weeks later, mice were euthanized and dissected. (**A**) Representative micro-CT X-ray images of explanted nodules show significant mineralized structure in each cell group of implants. (**B**) Incidences of mineralized nodule formation in each group. *P < 0.05; Z proportion score. (**C**) Quantification of mineralized nodule volume and micro-CT total intensity in each group. All data represent with interleaved scatter plots with mean ± SD (multiple *t* test; *P < 0.05; **P < 0.01; ^#^*P* < 0.001). (**D**) Serial cryosections were made in each explanted cell nodules. Mineralized structures were visualized using Alizarin red S and Von Kossa staining. Newly formed mineralized structures were visualized by Aniline Blue staining.

The original Article has been corrected.

