# Supplementary material for: The role of vesicle trafficking genes in osteoblast differentiation and function
Source: Sci Rep. 2023 Sep 26;13:16079. doi: 10.1038/s41598-023-43116-8 (PMC10522589; doi:10.1038/s41598-023-43116-8)
Supplement: Supplementary file 1 — Supplementary Information 1. [file 41598_2023_43116_MOESM1_ESM.docx]

**Supplementary Information**

The role of vesicle trafficking genes in osteoblast differentiation and function

Hui Zhu^1^, Yingying Su^1^, Jamie Wang^1^, Joy Wu^1, *^

^1^Division of Endocrinology, Stanford University School of Medicine, Stanford, CA, USA

* Corresponding author

Email: [jywu1@stanford.edu](mailto:jbaker@stanford.edu)

**Supplementary Figures**

**
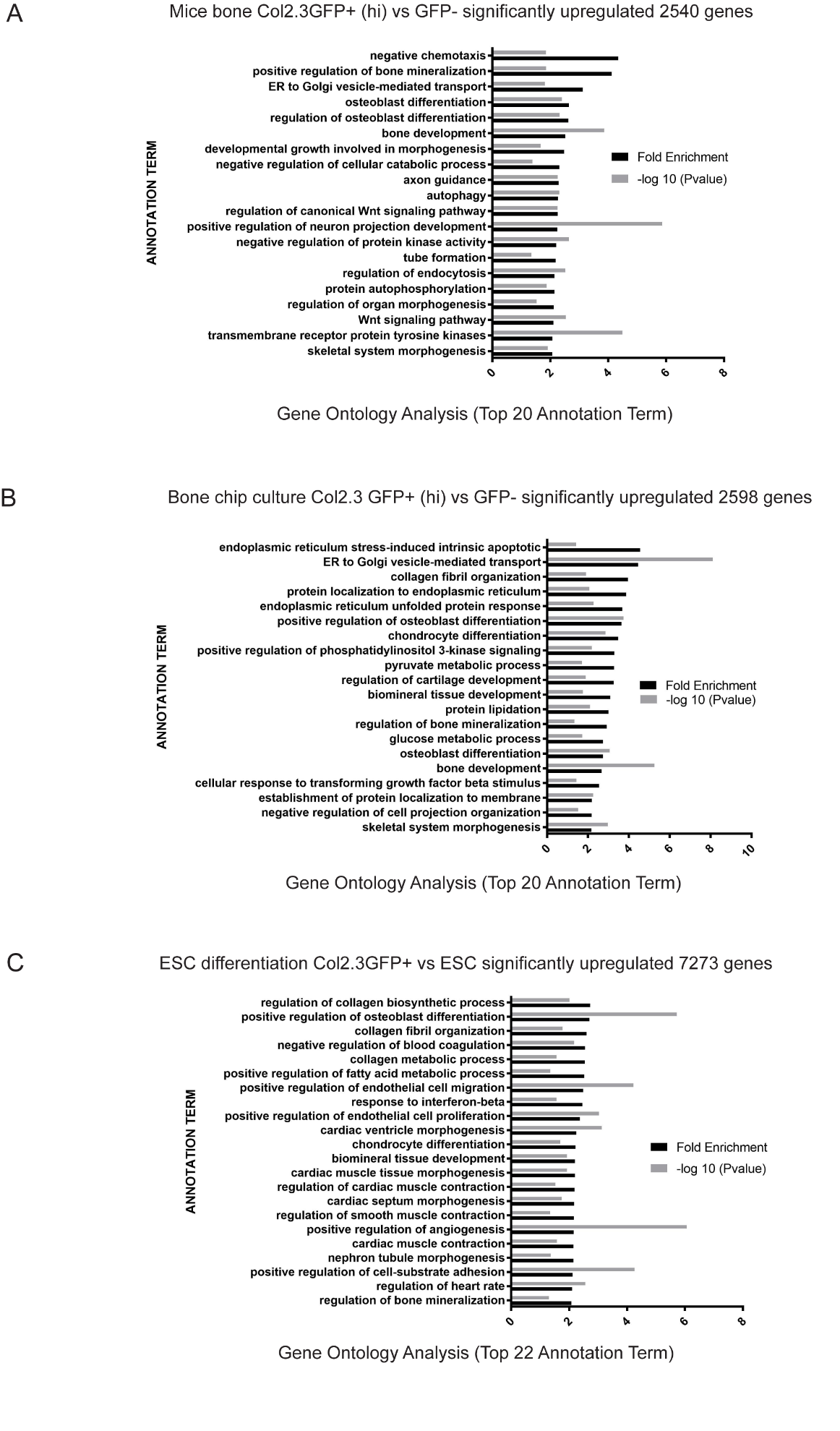
**

Fig. S1. Significantly upregulated genes in Col2.3GFP+ populations are enriched in osteoblast differentiation, bone development, bone mineralization and ossification annotation categories.


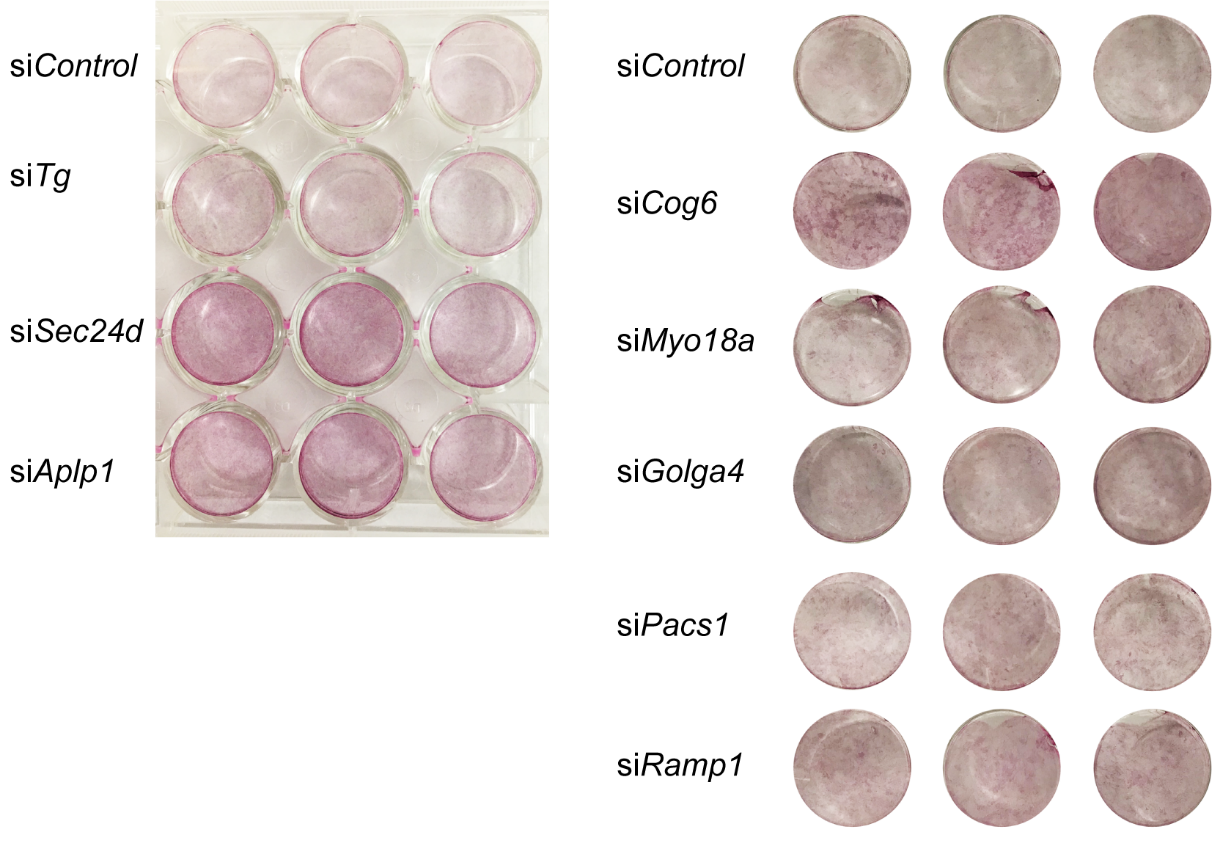


Fig. S2. Transient knockdown of vesicle trafficking genes increases alkaline phosphatase activity in MC3T3-E1 osteogenic differentiation. Seven days after osteogenic differentiation, representative alkaline phosphatase staining images in control and trafficking gene knockdown MC3T3-E1 osteoblast differentiation.


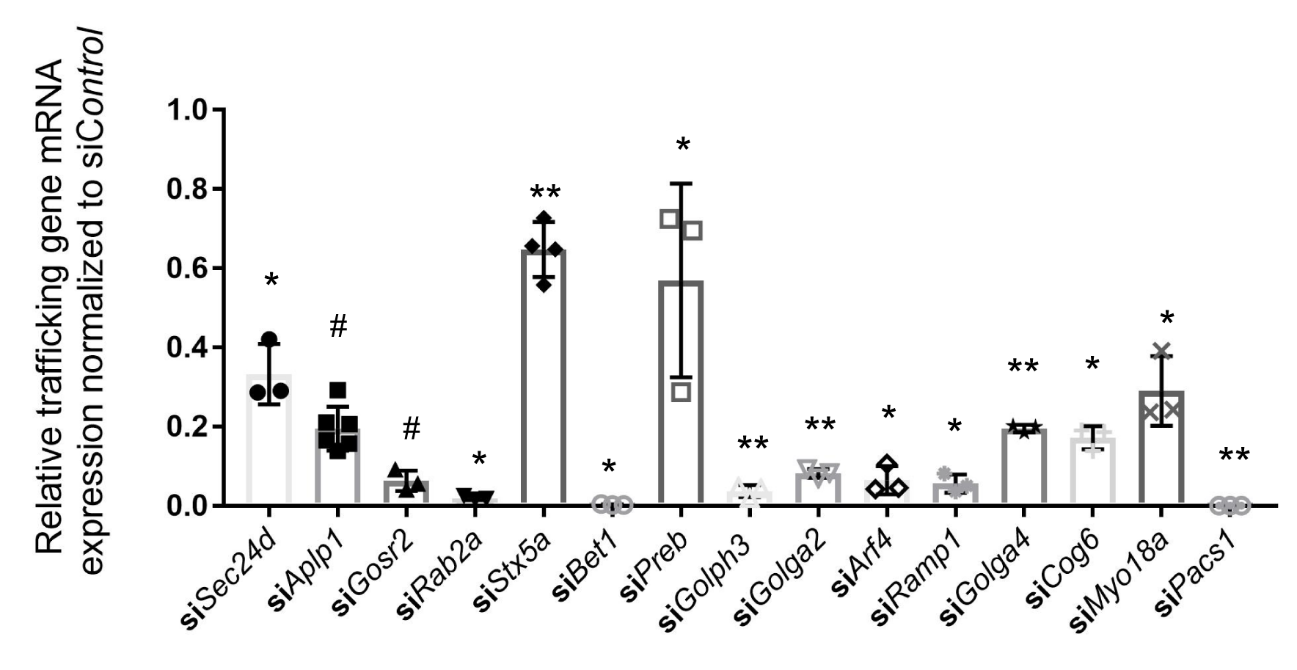


Fig. S3. Trafficking gene mRNA levels decrease after siRNA transfection. Three days after siRNA transfection, relative target trafficking gene mRNA expression in control and trafficking gene knockdown MC3T3-E1 cells were analyzed by qRT-PCR. Y axes show relative target trafficking gene mRNA expression in trafficking gene knockdown normalized to si*Control*. All data represent with scatter plots with mean ± SD (multiple *t* test; *, *P*<0.05; **, *P*<0.01; #, *P*<0.001; relative to si*Control*).


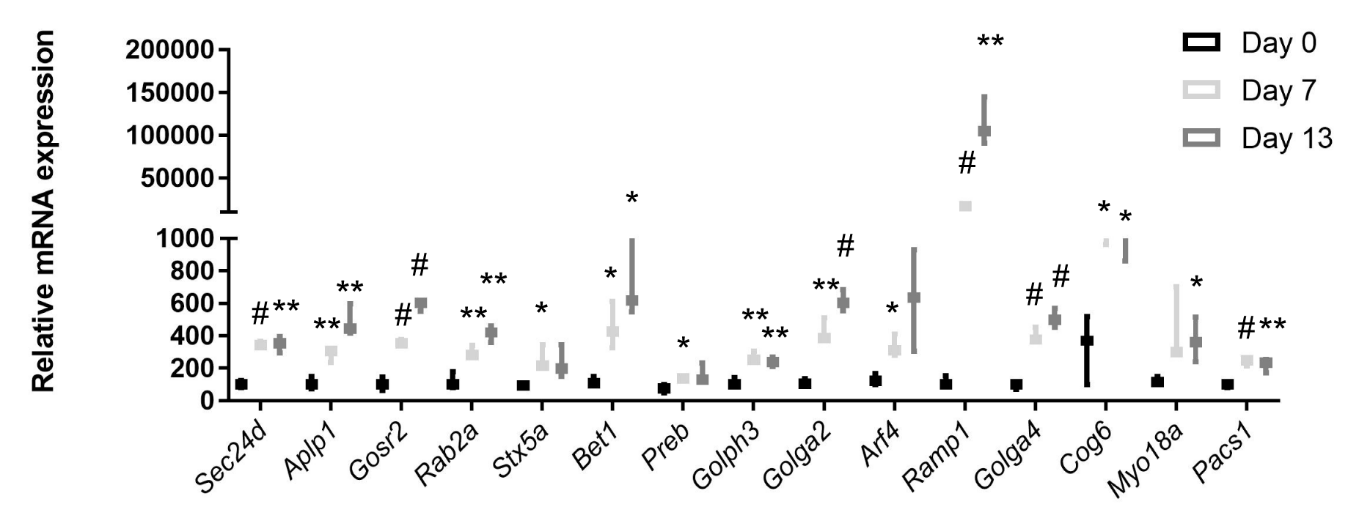


Fig. S4. Trafficking gene expression increases with osteogenic differentiation. Seven and thirteen days after MC3T3-E1 cell osteogenic differentiation, qRT-PCR analysis shows relative trafficking gene mRNA levels. All data represent with Interleaved box-whiskers plots (N=3; multiple *t* test; *, *P*<0.05; **, *P*<0.01; #, *P*<0.001; relative to Day 0).


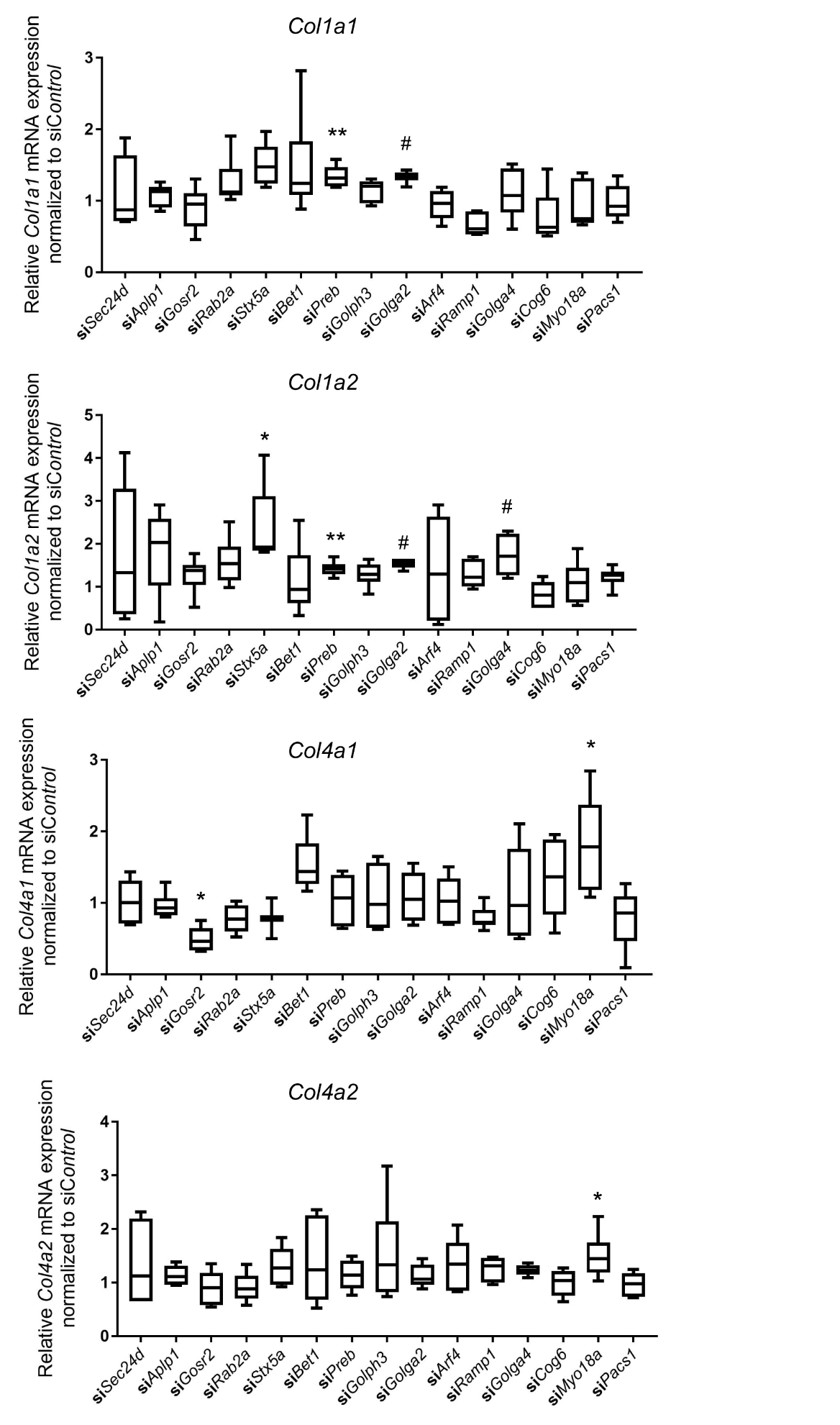


Fig. S5. Expression of collagen genes with trafficking gene knockdown in MC3T3-E1 osteogenic differentiation. Ten days after osteogenic differentiation, relative *Col1a1*, *Col1a2*, *Col4a1* and *Col4a2* mRNA levels in control and trafficking gene knockdown MC3T3-E1 cell differentiation were analyzed by qRT-PCR. All data represent with box-whiskers plots (N=6-9; multiple *t* test; *, *P*<0.05; **, *P*<0.01; #, *P*<0.001; relative to si*Control*).


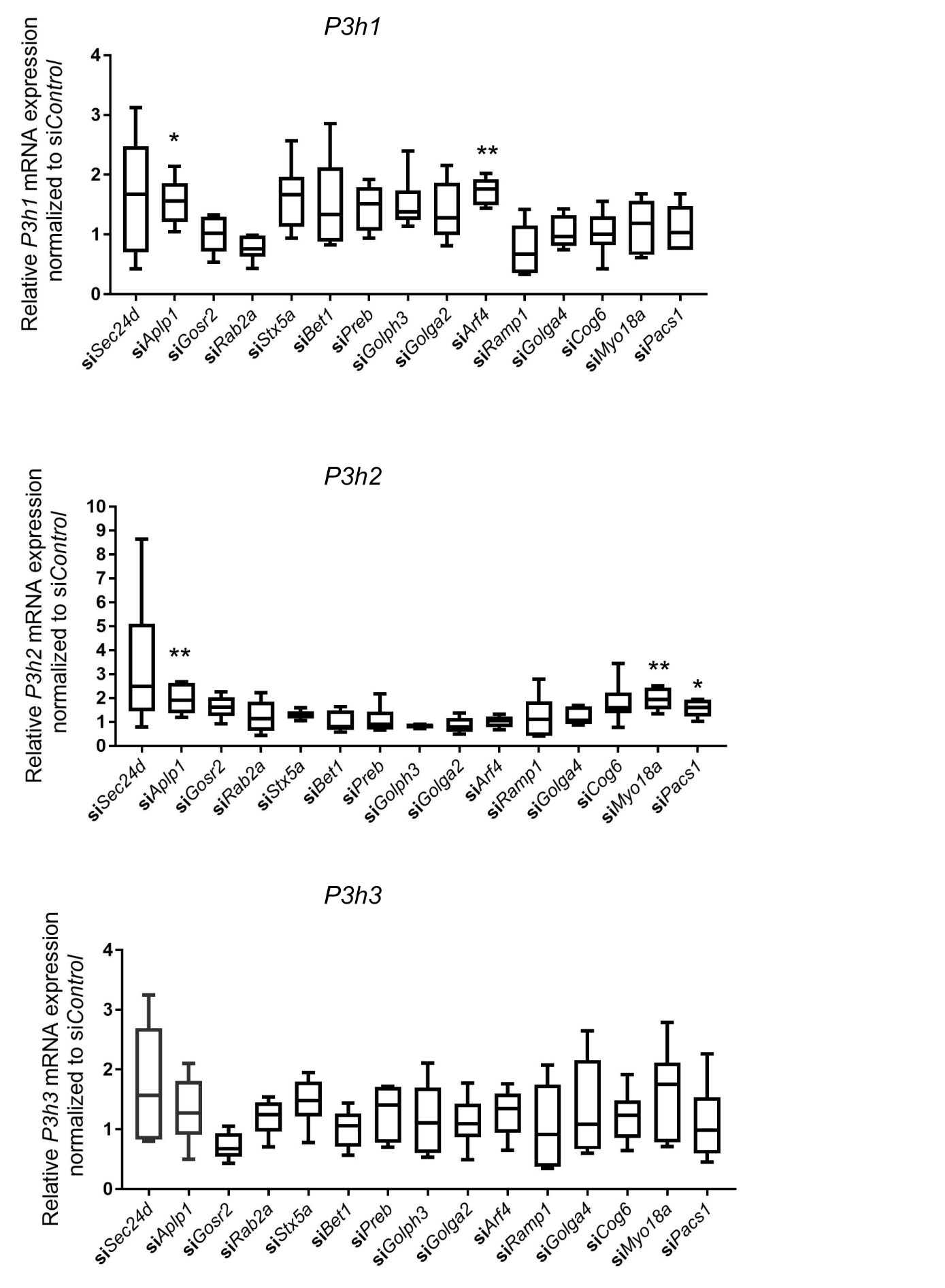


Fig. S6. Expression of collagen prolyl 3-hydroxylase *P3h* isoenzymes with trafficking gene knockdown in MC3T3-E1 osteogenic differentiation. Ten days after osteogenic differentiation, relative *P3h1*, *P3h2 and P3h3* mRNA expression in control and trafficking gene knockdown MC3T3-E1 cell differentiation were analyzed by qRT-PCR. Y axes show relative *P3h1*, *P3h2 and P3h3* mRNA expression in trafficking gene knockdown normalized to si*Control*. All data represent with box-whiskers plots (N=6-9; multiple *t* test; *, *P*<0.05; **, *P*<0.01; #, *P*<0.001; relative to si*Control*).


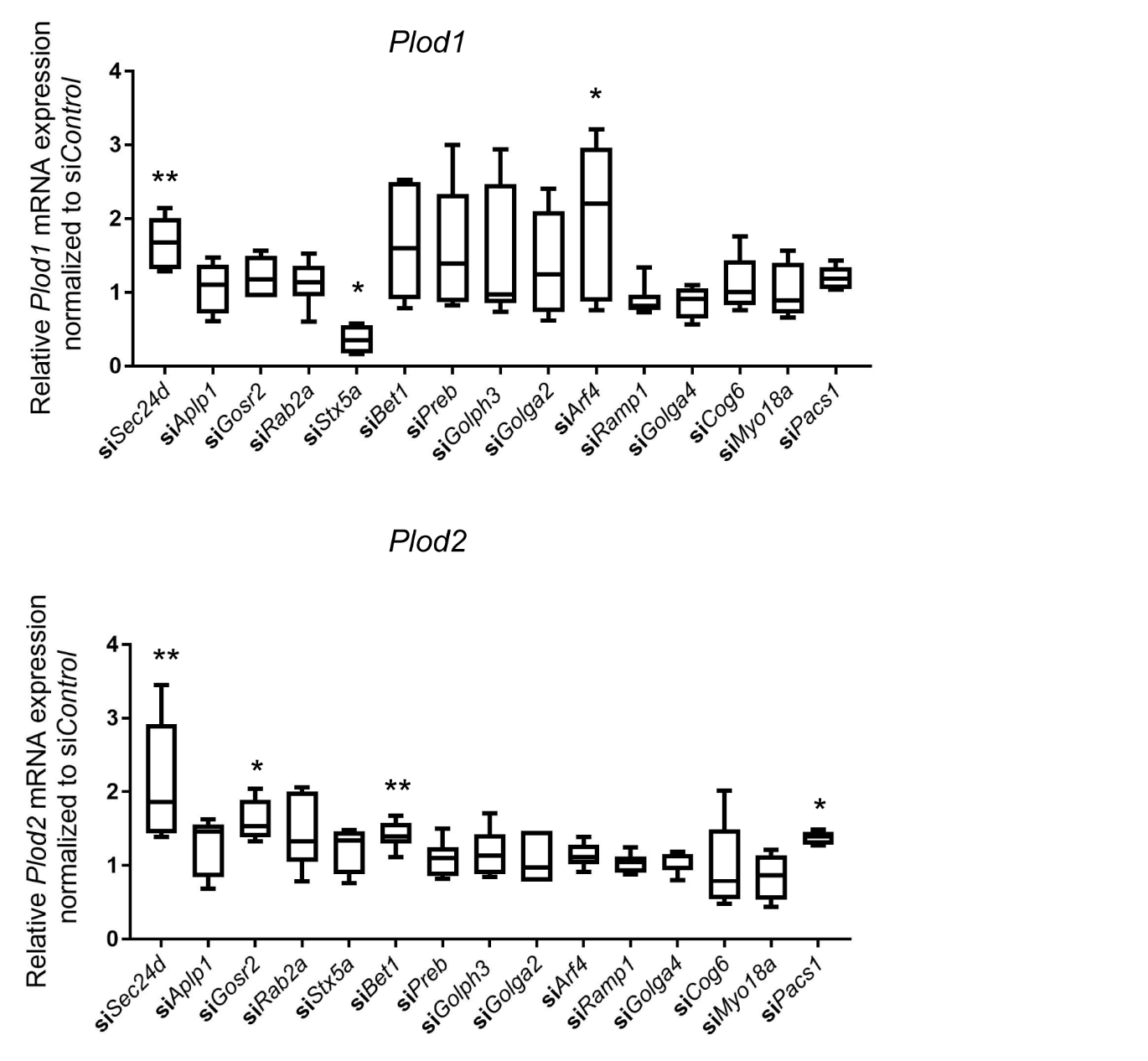


Fig. S7. Expression of collagen lysine hydroxylase *Plod* isoenzymes with trafficking gene knockdown in MC3T3-E1 osteogenic differentiation. Ten days after osteogenic differentiation, relative *Plod1*, and *Plod2* mRNA expression in control and trafficking gene knockdown MC3T3-E1 cell differentiation were analyzed by qRT-PCR. Y axes show relative *Plod1* and *Plod2* mRNA expression in trafficking gene knockdown normalized to si*Control*. All data represent with box-whiskers plots (N=6-9; multiple *t* test; *, *P*<0.05; **, *P*<0.01; #, *P*<0.001; relative to si*Control*).


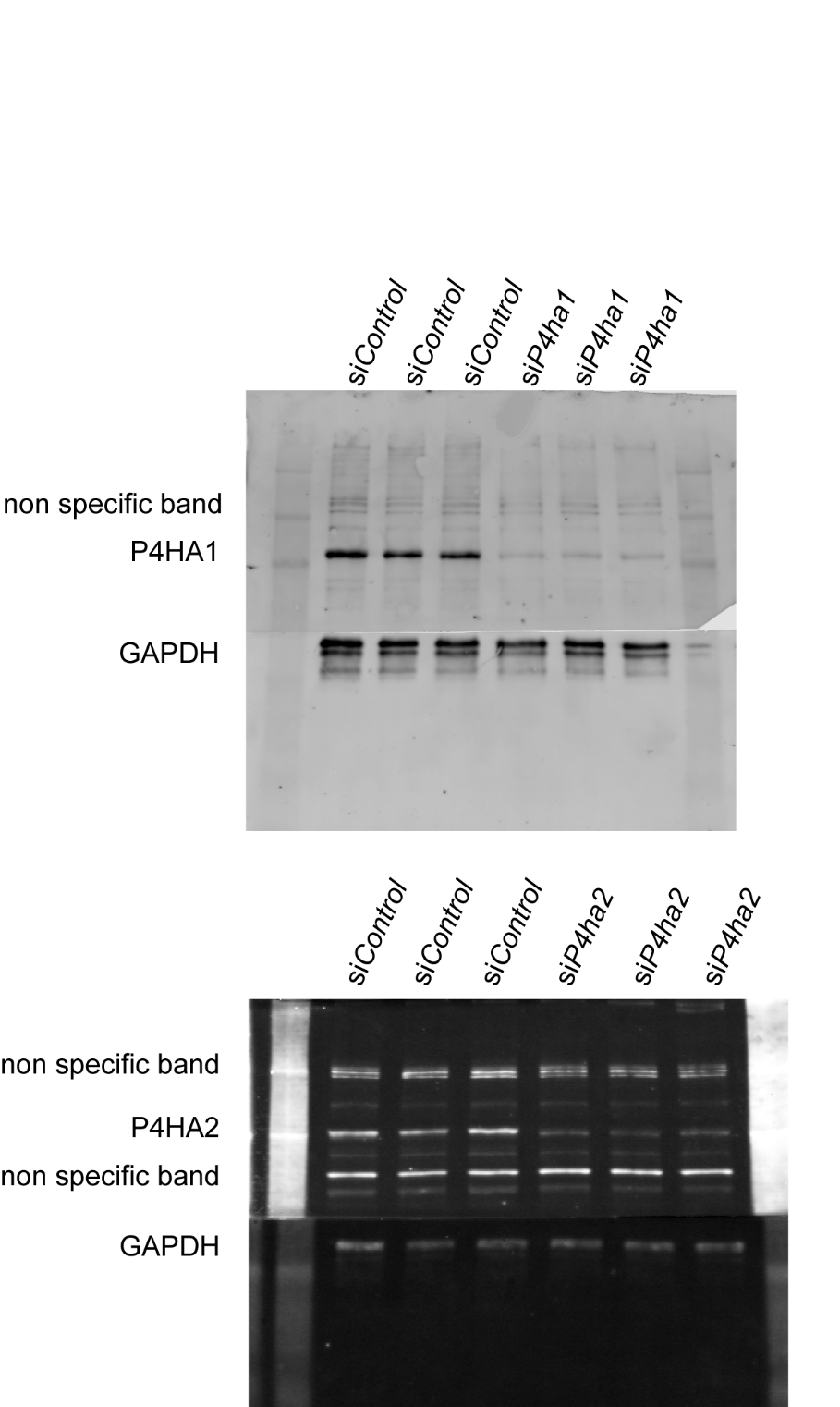


Fig. S8. P4HA1 and P4HA2 protein levels decrease with knockdown *P4ha1* and *P4ha2*. Three days after siRNA transfection, Western-blot analysis shows P4HA1 and P4HA2 protein depleted in control and *P4ha1* or *P4ha2* knockdown MC3T3-E1 cells.


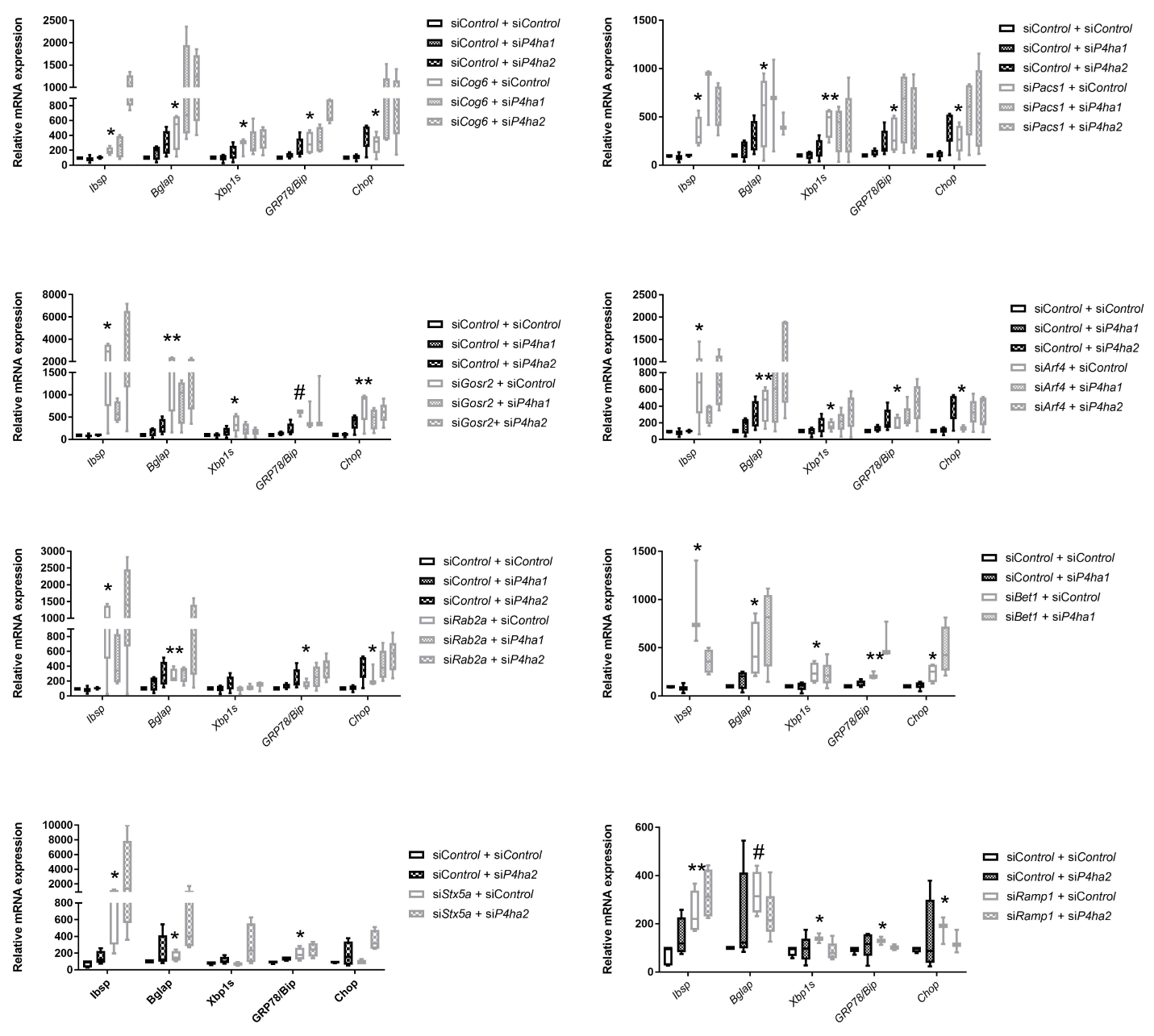


Fig. S9. Concurrently knockdown *P4ha* with vesicle trafficking gene do not affects the expression of osteoblast marker and ER stress sensor. Ten days after osteogenic differentiation, relative *Ibsp*, *Bglap*, *Xbp1s*, *GRP78/Bip* and *Chop* mRNA expression in control and trafficking gene with/without concurrently *P4ha1* and *P4ha2* knockdown in MC3T3-E1 cell differentiation were analyzed by qRT-PCR. All data represent with box-whiskers plots (N=6-9; multiple *t* test; *, *P*<0.05; **, *P*<0.01; #, *P*<0.001; relative to si*Control* + si*Control*).


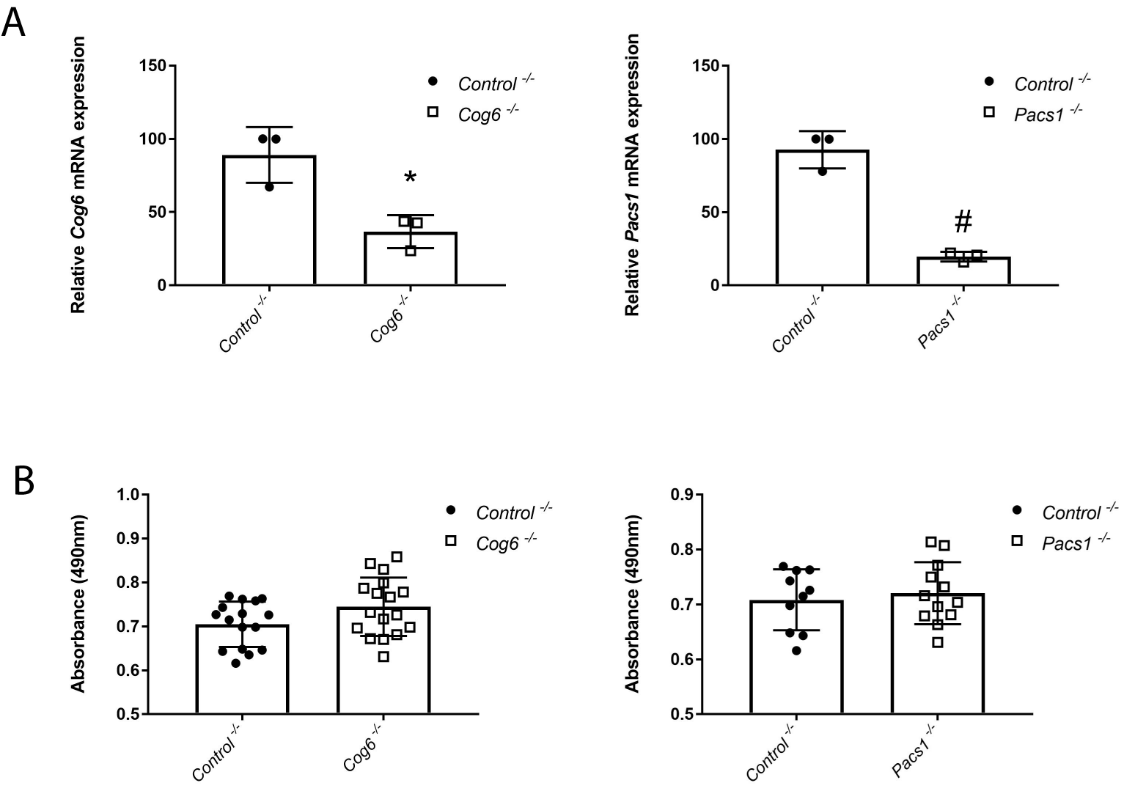


Fig. S10. *Cog6*^-/-^ and *Pacs1*^-/-^ MC3T3-E1 CRISPR line characterization. *Cog6***^-/-^,** *Pacs1***^-/-^** MC3T3-E1 CRISPR lines and control CRISPR line were subjected to osteogenesis for ten days. (*A*) qRT-PCR analysis shows significantly decreased mRNA in *Cog6***^-/-^** and *Pacs1***^-/-^** MC3T3-E1 CRISPR lines compared to control CRISPR line. (*B*) MTS cell proliferation assay shows that *Cog6* or *Pacs1* depletion doesn’t change cell proliferation rate. All data represent with scatter plots with mean ± SD (multiple *t* test; *, *P*<0.05; **, *P*<0.01; #, *P*<0.001; relative to *Control***^-/-^**).


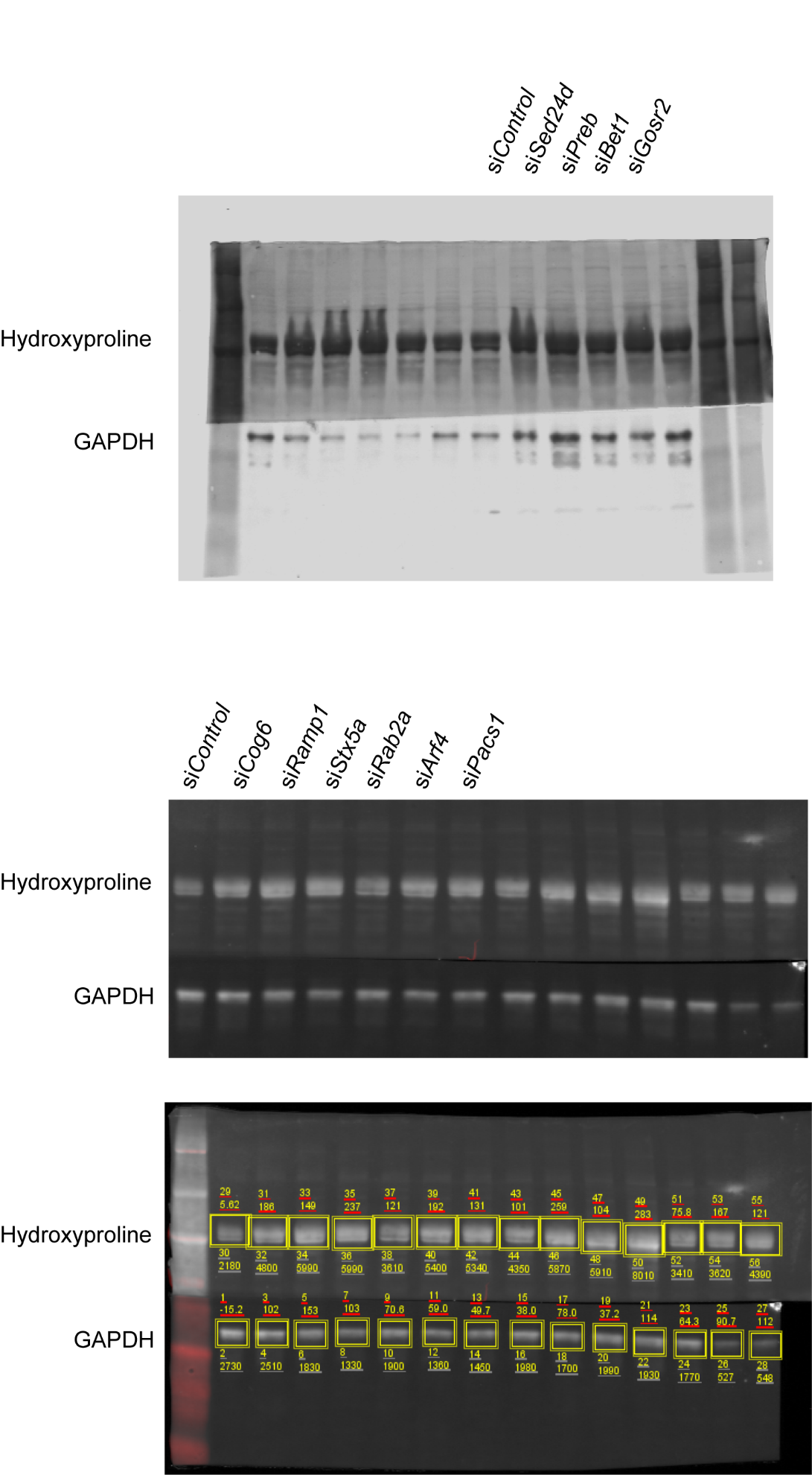


Fig. S11. Full gel blot related to Figure 5B. Hydroxyproline protein levels increase with vesicle trafficking gene knockdown in MC3T3-E1 osteogenic differentiation. Ten days after osteogenic differentiation, Western-blot analysis shows Hydroxyproline and GAPDH protein expression in control and trafficking gene knockdown MC3T3-E1 cell differentiation.

**Supplementary Table**

qRT-PCR primer sequences:

| Gene | Forward primer (5’-3’) | Reverse primer (5’-3’) |
| --- | --- | --- |
| *Sec24d* | GGAGAGGTCTTTGTTCCTTTGTT | GTCTCTGTTCTTGAGCTTCCC |
| *Aplp1* | GTGGGCGTCTAACCCTTCAC | CTGCGGGTCCAGAAGACAG |
| *Gosr2* | CCCCTATACCAACAAACGAACA | GGCTCCTTGCTTGACAAAATCT |
| *Rab2a* | GCGACACAGGTGTTGGTAAAT | CATCAATCGTTATCATCCGAGCA |
| *Stx5a* | CGGAAACGCTACGGATCTAAG | CAGGGGACAGAACCTGTGT |
| *Bet1* | ACTATGGCTATGCCAATACTGGT | CTCCCTCTGGACAAAATCTTCAG |
| *Preb* | GCTTCGGATAGACCCCAAGAC | CAAGCGCCAAATTCATGGTG |
| *Golph3* | ACAAGGGCGACTCCAAGGA | ATGATGTGTAACCCTCTCGGT |
| *Golga2* | TGTCACAGAACCGAGAGCTGA | GCTGCTCCTGTAGACCCTG |
| *Arf4* | CTGGCAAGACGACAATTCTGT | CCACAAAAATGAGACCCTGGGTA |
| *Ramp1* | CCTCTGCTTACCTCTGAGATTG | ATCTGTGCAGTCTTCCTTGGAGT |
| *Golga4* | CGGGGCTCATCAGCGAAAA | AGACTGGACTATGCGGACCT |
| *Cog6* | CAGAGCCTACGCGGAAGTG | AGGTGACCTATGAAGGATGCT |
| *Myo18a* | TGACAGCAATCGGGGTAGC | GCCAGTGAACCGAACTTGG |
| *Pacs1* | GACTGGCTGGGTTACATGC | GCGACTGAATAGGTCTCTCCA |
| *Ibsp* | TACCGGCCACGCTACTTTCTTTAT | GACCGCCAGCTCGTTTTCATCC |
| *Bglap* | TCTCTCTGCTCACTCTGCTGGCC | TTTGTCAGACTCAGGGCCGC |
| *Col1a1* | CACCCTCAAGAGCCTGAGTC | GTTCGGGCTGATGTACCAGT |
| *Col1a2* | TCGGGCCTGCTGGTGTTCGTG | TGGGCGCGGCTGTATGAGTTCTTC |
| *Col4a1* | CTGGAGAAAAGGGCCAGAT | TCCTTAACTTGTGCCTGTCCA |
| *Col4a2* | CGACCGAGTGCGGTTCAAA | ACCGCAGGGCACATCCAACT |
| *Xbp1s* | GAGTCCGCAGCAGGTG | GTGTCAGAGTC-CATGGGA |
| *GRP78/BiP* | CATGGTTCTCACTAAAATGAAAGG | GCTGGTACAGTAACAACTG |
| *Chop* | CTGCCTTT CACCTTGGAGAC | CGTTTCCTGGGGATGAGATA |
| *P4ha1* | AGCCACCATTTCAAACCCAGT | GCCAAGCACTTTTGCTAATTCTG |
| *P4ha2* | CAGGTACTATGATGTGATGTCCG | AAAGGGTCGCCTTGAGAAGTC |
| *P3h1* | CCCCCAGCCTACACGTTCCG | TGCCCCCTGGTGACAGCCTTC |
| *P3h2* | ATGCTAAAACCGTGACTGCC | CGCTCCAGTTCTCGGTAAAG |
| *P3h3* | AGAGGGCCTACTACCAGTTG | GTGTTGGGTTTGCCACAAAG |
| *Plod1* | GAAGGATGACGCCAAGCTAGA | TGAAGAACTGAGCTGAACGCT |
| *Plod2* | GAGAGGCGGTGATGGAATGAA | ACTCGGTAAACAAGATGACCAGA |
